# The dehydrogenase region of the NADPH oxidase component Nox2 acts as a protein disulfide isomerase (PDI) resembling PDIA3 with a role in the binding of the activator protein p67^*phox*^

**DOI:** 10.3389/fchem.2015.00003

**Published:** 2015-02-04

**Authors:** Edna Bechor, Iris Dahan, Tanya Fradin, Yevgeny Berdichevsky, Anat Zahavi, Aya Federman Gross, Meirav Rafalowski, Edgar Pick

**Affiliations:** The Julius Friedrich Cohnheim Laboratory of Phagocyte Research, Department of Clinical Microbiology and Immunology, Sackler School of Medicine, Tel Aviv UniversityTel Aviv, Israel

**Keywords:** protein disulfide isomerase, superoxide, NADPH oxidase, Nox2, p67^*phox*^, synthetic peptides, Cys-Gly-Cys triad, disulfide bonds

## Abstract

The superoxide (O^·−^_2_)-generating NADPH oxidase of phagocytes consists of a membrane component, cytochrome *b*_558_ (a heterodimer of Nox2 and p22^*phox*^), and four cytosolic components, p47^*phox*^, p67^*phox*^, p40^*phox*^, and Rac. The catalytic component, responsible for O^·−^_2_ generation, is Nox2. It is activated by the interaction of the dehydrogenase region (DHR) of Nox2 with the cytosolic components, principally with p67^*phox*^. Using a peptide-protein binding assay, we found that Nox2 peptides containing a ^369^CysGlyCys^371^ triad (CGC) bound p67^*phox*^ with high affinity, dependent upon the establishment of a disulfide bond between the two cysteines. Serially truncated recombinant Nox2 DHR proteins bound p67^*phox*^ only when they comprised the CGC triad. CGC resembles the catalytic motif (CGHC) of protein disulfide isomerases (PDIs). This led to the hypothesis that Nox2 establishes disulfide bonds with p67^*phox*^ via a thiol-dilsulfide exchange reaction and, thus, functions as a PDI. Evidence for this was provided by the following: (1) Recombinant Nox2 protein, which contained the CGC triad, exhibited PDI-like disulfide reductase activity; (2) Truncation of Nox2 C-terminal to the CGC triad or mutating C369 and C371 to R, resulted in loss of PDI activity; (3) Comparison of the sequence of the DHR of Nox2 with PDI family members revealed three small regions of homology with PDIA3; (4) Two monoclonal anti-Nox2 antibodies, with epitopes corresponding to regions of Nox2/PDIA3 homology, reacted with PDIA3 but not with PDIA1; (5) A polyclonal anti-PDIA3 (but not an anti-PDIA1) antibody reacted with Nox2; (6) p67^*phox*^, in which all cysteines were mutated to serines, lost its ability to bind to a Nox2 peptide containing the CGC triad and had an impaired capacity to support oxidase activity *in vitro*. We propose a model of oxidase assembly in which binding of p67^*phox*^ to Nox2 via disulfide bonds, by virtue of the intrinsic PDI activity of Nox2, stabilizes the primary interaction between the two components.

## Introduction

Oxygen-derived radicals are the principal mediators responsible for the killing of pathogenic microorganisms by phagocytes. All oxygen radicals produced by phagocytes are derived from the superoxide anion (O^·−^_2_), generated by the NADPH-derived one-electron reduction of molecular oxygen. The process is catalyzed by a membrane-imbedded 91 kDa flavoprotein, known as Nox2, which is associated with a second protein of 22 kDa (p22^*phox*^), to form the flavocytochrome *b*_558_ heterodimer. Nox2 is 570 residues-long and comprises six transmembrane α-helices linked by three outside-facing loops and two cytosol-facing loops, and a cytosolic segment, extending from residue 288 to 570. Nox2 contains all redox stations supporting the flow of electrons from NADPH to oxygen. These are an NADPH-binding site and non-covalently bound FAD, present in the cytosolic segment, and two non-identical hemes present in the third and fifth membrane helices (reviewed in Quinn and Gauss, 2004).

In the resting phagocyte, no electron flow occurs along the redox centers in Nox2. The initiation of the electron flow is mediated by a conformational change in Nox2, resulting from the interaction with regulatory proteins present in the cytosol. The regulatory cytosolic components are p47^*phox*^, p67^*phox*^, p40^*phox*^, and the small GTPase Rac (1 or 2) and they translocate to the membrane environment of Nox2, to generate the NADPH oxidase complex (briefly, oxidase), a process known as oxidase assembly (reviewed in Groemping and Rittinger, 2005). In the intact cell, p47^*phox*^, p67^*phox*^, and Rac are all required for the induction of O^·−^_2_ production but it is yet unsettled whether direct interaction of all components with Nox2 is required. There is evidence for p67^*phox*^ being the key component responsible for the causation of a conformational remodeling of Nox2 (Kreck et al., 1996; Gorzalczany et al., 2000). Major unsolved issues are the identities of region(s) in Nox2 and p67^*phox*^ participating in the interaction among the two. It has been found that an “activation domain” comprising residues 199–210 (Han et al., 1998) or a wider region, extending from residue 190 to 208 (Sumimoto, 2008) in p67^*phox*^ is essential for oxidase activation but not for the actual p67^*phox*^–Nox2 interaction. Experimental evidence related to the latter suggests that the binding site for Nox2 is located N-terminal to residue 198 (Dang et al., 2002) and, most likely, between residues 187 and 198 or 194 and 198 (Federman Gross et al., 2012).

The cytosolic segment of Nox2 is also known as the dehydrogenase region (DHR) by virtue of the fact that it contains the NADPH- and FAD-binding sites and is homologous to the prokaryotic protein ferredoxin-NADP^+^ reductase. The DHR comprises binding sites for p47^*phox*^ and Rac but, so far, there is no solid evidence for the identity of the binding site(s) for p67^*phox*^. We approached this question by applying the “peptide walking” methodology (Joseph and Pick, 1995; Dahan et al., 2012; Dahan and Pick, 2012). Overlapping 15-mer peptides, corresponding to the DHR of Nox2 (residues 288–570) were attached to 96-well plates and reacted with recombinant 6His-p67^*phox*^ in the fluid phase; peptide-bound p67^*phox*^ was detected by peroxidase-conjugated anti-polyHis antibody. It was found that p67^*phox*^ binds preferentially to two peptides, corresponding to residues 357–371 (termed Nox2 peptide 24) and 369–383 (termed Nox2 peptide 28) (Dahan and Pick, manuscript in preparation). The peptides share a ^369^CysGlyCys^371^ (CGC) triad, located at the C-terminus of peptide 24 and the N-terminus of peptide 28. The CGC triad is present in the DHR of Nox2 of all species, down to amphibians, and is absent in Nox1, 3, 4, and 5 (Kawahara et al., 2007). Peptides derived from Nox4, corresponding to Nox2 peptides 24 and 28 by sequence alignment but lacking the CGC triad, did not bind p67^*phox*^. It is of interest that Nox4 generates oxygen radicals constitutively, without a requirement for cytosolic activators, such as p67^*phox*^ (Bedard and Krause, 2007).

Replacing C369 or C371 with Arg or Ser abolished binding of p67^*phox*^ to peptides 24 and 28. A 369Cys to Arg mutation in Nox2 causes chronic granulomatous disease (CGD) of the X91^+^ form, with normal expression of Nox2 but impaired production of O^·−^_2_, impaired translocation of cytosolic components, and low FAD binding (Leusen et al., 2000; Debeurme et al., 2010).

We next found that the introduction of an intramolecular disulfide bond between C369 and C371 in Nox2 peptides 24 and 28 resulted in a marked increase in the binding of p67^*phox*^. Reduction of the disulfide bond and alkylation of the reduced thiols totally abolished binding of p67^*phox*^ (Fradin et al., 2011, 2012; Pick, 2012; Fradin and Pick, manuscript in preparation). An important observation was that enhanced binding of p67^*phox*^ was evident only when the disulfide bond was established between two non-adjacent cysteines and between cysteines present in the same peptide; when the CGC triad was replaced by CCG and a disulfide bond established between the adjacent cysteines or the disulfide bond linked C369 or C371 on two peptides, forming a dimer, no enhanced binding of p67^*phox*^ was found.

These observations are to be related to a large body of early work by several groups showing that thiol alkylating agents interfere with oxidase activation in intact phagocytes and in *in vitro* systems. Thus, *N*-ethylmaleimide (NEM) inhibited oxidase activation in intact phagocytes, if added before activation (Cohen and Chovaniec, 1978; Yamashita et al., 1984) and *in vitro* (Shpungin et al., 1989) and was shown to act on a membrane component (Shpungin et al., 1989). Similar results were obtained with 4-(hydroxymercuri)benzoic acid [HMBA, known in the past as *p*-chloromercuribenzoate (PCMB)]. A solubilized membrane-associated fraction, prepared from stimulated neutrophils, was inactivated by HMBA (Bellavite et al., 1983; Gabig and Lefker, 1984) and macrophage membrane liposomes treated with HMBA were inactive in a cell-free oxidase activation system (Pick et al., 1987). Less defined evidence exists for cysteines being the target for the oxidase inhibitors apocynin, VAS2870, and ebselen, and the specific components affected were not rigorously identified.

More recent testimony for the involvement of Nox2-localized cysteines in oxidase function was provided by the discovery of the marked inhibitory effect of phenylarsine oxide (PAO), an agent which reacts with vicinal dithiols to form a disulfide-like complex (van Iwaarden et al., 1992). PAO was found to inhibit oxidase activation in neutrophils and *in vitro*, when preincubated with the membrane fraction, a reaction that was reversed by reducing agents (Le Cabec and Maridonneau-Parini, 1995). PAO was proposed to act on the pair of cysteines 369 and 371 in Nox2 (Doussiere et al., 1998). More support for the role of vicinal cysteines in Nox2 in oxidase activation was offered by the inhibitory effect of the fungal toxin gliotoxin (Waring and Beaver, 1996), which contains a bridged disulfide ring capable of forming mixed disulfides with thiol-containing proteins. Gliotoxin was reported to inhibit oxidase activation in neutrophils (Tsunawaki et al., 2004) and *in vitro*, with the vicinal cysteines 369 and 371 in Nox2, considered as likely targets (Nishida et al., 2005). Gliotoxin did not affect Nox4 (Serrander et al., 2007), in good agreement with the facts that Nox4 does not contain the CGC triad and does not require cytosolic assistance for generation of reactive oxygen species (ROS).

On considering our results on the binding of p67^*phox*^–Nox2 peptides containing the CGC triad, in the disulfide form, and the accumulated evidence in support of a role for these cysteines in oxidase assembly, we reasoned that, at a certain stage in the process of oxidase assembly, the intramolecular disulfide bond in Nox2 might be converted to an intermolecular disulfide bond between Nox2 and cysteines in p67^*phox*^ by a thiol—disulfide exchange reaction. It is likely that the primary interaction between the Nox2 DHR and p67^*phox*^ is based on specific binding sites in the two partners and does not involve disulfide bonds. The establishment of disulfide bonds between cysteines in the Nox2 CGC triad and cysteines in p67^*phox*^ is a secondary event with a stabilizing role.

It is our hypothesis that Nox2 serves as an endogenous protein disulfide isomerase (PDI), when the cysteines in the CGC triad are in the disulfide form. PDIs are multi-domain proteins belonging to the thioredoxin superfamily (reviewed in Collet and Messens, 2010) and to the PDI gene family, which comprises 21 members, varying in size, domain composition and tissue expression (reviewed in Ellgaard and Ruddock, 2005; Appenzeller-Herzog and Ellgaard, 2008; Galligan and Petersen, 2012; Ali Khan and Mutus, 2014). PDIs can catalyze thiol—disulfide oxidation and reduction and disulfide rearrangement (isomerization) and also function as chaperones. PDIs contain two thioredoxin-like catalytic domains, with a characteristic CXXC active site motif. This is CGHC, in most PDIs, as opposed to the CGPC sequence, typical of thioredoxin.

The proposal that Nox2 acts as a PDI is backed by the following body of evidence: (a) The CGC triad closely mimics the two CGHC catalytic motifs of PDI; thus CGC has a disulfide reduction potential (E°′) of −167 mV, which is very close to that of the CGHC motif of PDI (−180 mV) (Woycechowsky and Raines, 2003); (b) The disulfide bond in CGC is relatively unstable with high loop opening (*k_o_*) and closing (*k_c_*) constants, a property favorable to PDI activity (Zhang and Snyder, 1989; Kersteen and Raines, 2003); (c) Small peptides CGC and RKCGC, in the disulfide form, can mimic the actions of PDI protein (Kersteen and Raines, 2003; Wang et al., 2011), and (d) The CGC triad resembles similar motifs appearing in a large family of eukaryotic and prokaryotic enzymes known as thiol-disulfide oxidoreductases, which contain one or more di-cysteine motifs and, sometimes, a flavin cofactor (Sevier and Kaiser, 2002).

In the present work we present the experimental evidence in support of the hypothesis that the DHR of Nox2 functions as a PDI, leading to establishment of disulfide bonds with p67^*phox*^ and assuring the stabilization of the NADPH oxidase complex. This conclusion is based on the use of a wide range of methodologies, comprising the generation of recombinant DHR of Nox2 and p67^*phox*^ and mutants of these, protein - protein and peptide—protein binding assays, an enzymatic PDI reductase assay, immunologic characterization, the use of PDI inhibitors, and bioinformatics.

## Materials and methods

### Synthetic peptides

Synthetic peptides corresponding to selected sequence segments of Nox2 were made by two companies: Mimotopes (Clayton, Victoria, Australia), and Bachem (Bubendorf, Switzerland). Peptides were 15-residues-long, with a biotin tag at the N-terminus (attached by a Ser-Gly-Ser-Gly spacer) and a C-terminal amide. Peptides were of a purity of at least 70%, documented by reversed phase chromatography, and the size, confirmed by MALDI-mass spectroscopy. Peptides were dissolved in a mixture of 75 parts 1-methyl-2-pyrrolidone and 25 parts water (v/v), to a concentration of 1.5 mM, to serve as stock solutions for further dilution, and kept frozen at −75°C in small aliquots. Working solutions were freshly prepared on the day of performing the experiments.

### Chemicals

Common laboratory chemicals, at the highest purity available, were purchased from Merck KGaA (Darmstadt, Germany), or Sigma-Aldrich (St. Louis, MO, USA). The following potential PDI inhibitors were used: phenylarsine oxide (Sigma-Aldrich); bacitracin (mixture of 9 bacitracins, mainly type A; Sigma-Aldrich); gliotoxin (from *Gliocladium fimbriatum*, Fermentek, Jerusalem, Israel); PDI inhibitor 16F16 (Sigma-Aldrich); ribonuclease A, with scrambled disulfide bonds from bovine pancreas (Sigma-Aldrich), rutin hydrate (Sigma-Aldrich), and PDI inhibitor III, PACMA 31 (Merck). Dieosin glutathione disulfide (DE-GGSG), used in the PDI reductase assay, was a kind gift of Dr. Bulent Mutus (University of Windsor, Ontario, Canada). Nickel Sepharose 6 Fast Flow was obtained from GE Healthcare Bio-Sciences AB, Uppsala, Sweden. Monobromobimane Fluoropure grade (mBBr) was obtained from Molecular Probes, Life Technologies, Thermo Fisher Scientific, Waltham, MA, USA.

### Recombinant PDI

Recombinant PDI A1 (human; >95% pure; Cat. No. enz-262), made in *E. coli*, comprising a 12-His tag at the N-terminus, and recombinant PDI A3 (human; >95% pure; Cat. No. enz-474), made in *E. coli*, comprising residues 25–505 and a 37-His tag at the N-terminus, were obtained from ProSpec-Tany TechnoGene (Ness Ziona, Israel).

### Recombinant cytosolic NADPH oxidase components

Recombinant p67^*phox*^, p47^*phox*^, and Rac1 (Q61L mutant), with an N-terminal 6His tag, were expressed in *E. coli* and purified on Nickel Sepharose, as described before (Mizrahi et al., 2010). Recombinant non-prenylated Rac1 was prenylated *in vitro* by recombinant mammalian geranylgeranyltransferase type I (a gift of Dr. Carolyn Weinbaum, Duke University), as described before (Gorzalczany et al., 2002).

### Generation of recombinant p67^*phox*^ with 4 Cys to Ser mutations

We generated recombinant p67^*phox*^(1–212), in which cysteines 40, 45, 121, and 165 were mutated to serine. To construct a 6His-tagged protein in which the four cysteines were concurrently changed to serine, we used a synthetic gene encoding p67^*phox*^ amino acids 1–212, comprising the four mutations in plasmid pIDTSmart AMP (Integrated DNA Technologies, Coralville, IA, USA). This was subcloned into the *Bam*HI-*Eco*RI restriction sites of plasmid pET-30a-His_6_, as described for wild-type p67^*phox*^ (Mizrahi et al., 2010). The mutant protein was expressed in *E. coli* and purified on Nickel Sepharose, essentially as described for wild-type p67^*phox*^. The only deviation from the procedure applied to wild-type p67^*phox*^ was the supplementation of all buffers used for washing the Nickel Sepharose beads, and for binding and eluting the protein, with 0.2% (v/v) Triton X-100 (Sigma-Aldrich), with the purpose of preventing hydrophobic interaction with Nickel Sepharose. Such interaction was more pronounced with the mutant protein.

### Phagocyte membranes

Phagocyte membranes were prepared from guinea pig peritoneal macrophages elicited by the injection of mineral oil (Bromberg and Pick, 1984). The membranes were solubilized in 40 mM n-octyl ß-D-glucopyranoside and reconstituted into liposomes by dialysis against detergent-free buffer as described previously (Shpungin et al., 1989). The specific cytochrome *b*_558_ heme content of membrane vesicles was measured by the difference spectrum of sodium dithionite-reduced *minus* oxidized samples (Pick et al., 1987).

### Preparation of NusA-Nox2 fusion proteins

Recombinant fusion proteins linking parts of the DHR of human Nox2 (residues 288–570) with the *E. coli* protein NusA were constructed in order to obtain soluble Nox2 preparations. Recombinant NusA was also made, to serve as a negative control. The method was based on the ability of NusA to markedly improve the solubility of the fusion partner while conserving biological activity (Davis et al., 1999). Details of the construction of the fusion proteins are described under “Construction of NusA-Nox2 fusion proteins” in Supplementary Material. Fusion proteins were constructed to contain C-terminal to NusA the following Nox2 truncations: residues 328–570, 357–570, 372–570, 387–570, 408–570, 444–570, and 462–570. In addition, the fusion protein NusA-Nox2(357–570) was subjected to mutagenesis, in which Cys^369^ and Cys^371^ were mutated to Arg. The mutants were constructed using the “QuickChange Site-Directed Mutagenesis Kit” (Agilent Technologies, Santa Clara, CA, USA). Plasmid pET-43a-Nox2(357–570) was used as a template. The integrity of the mutant genes was confirmed by DNA sequencing. The fusion proteins and NusA were expressed in *E. coli* and purified on Nickel Sepharose, as described for the recombinant cytosolic NADPH oxidase components (Mizrahi et al., 2010). The NusA-Nox2 fusion proteins and NusA were further purified by fast protein liquid chromatography (FPLC) gel filtration on a HiLoad 16/60 Superdex 200 prep grade column (GE Healthcare Bio-Sciences AB), as described before (Mizrahi et al., 2010). This procedure separated a polymeric fraction from a homodimer fraction (197 kDa in size for NusA-Nox2(357–570), and 145 kDa, for NusA). The purified homodimers were used in enzymatic and immunologic assays.

### Determination of protein concentration and purity

The protein concentration of the recombinant proteins was measured by the method of Bradford (1976), modified for use with 96-wells microplates, using Bio-Rad protein assay dye reagent concentrate (Bio-Rad Laboratories, Hercules, CA, USA) and bovine γ-globulin as a standard. The level of purity of the recombinant proteins was assessed by SDS-PAGE analysis.

### SDS-page

SDS-PAGE was performed in a XCell *SureLock* Mini-Cell, using precast 12%, 1-mm thick, 10 well, NuPAGE Bis-Tris gels, and NuPAGE MOPS SDS running buffer. Run was at a constant voltage of 200 V for 50 min. All items were obtained from Novex, Life Technologies, Carlsbad, CA, USA, and the manufacturer's instructions were followed. Molecular weight standards (Precision Plus, All Blue, range 250–10 kDa, prestained) were purchased from Bio-Rad. The gels were stained by Instant Blue (Expedeon, Harston, UK).

### Antibodies

The following anti-human Nox2 antibodies were obtained from Santa Cruz Biotechnology (Santa Cruz, CA, USA): anti-Nox2 54.1 (mouse monoclonal, recognizing epitope 381–390 in the cytosolic segment of Nox2; Burritt et al., 1995; Baniulis et al., 2005); anti-Nox2 NL7 (mouse monoclonal, recognizing epitope 498–506 in the cytosolic segment of Nox2; Burritt et al., 2003), and anti-Nox2 C-15 (goat polyclonal raised against a peptide in the C-terminal region of Nox2; sequence is unknown). The following anti-human PDIA3 antibodies were used in this study: anti-PDIA3 H-220 (rabbit polyclonal, raised against residues 108–207; Santa Cruz); anti-PDIA3 HPA003230 (rabbit polyclonal, raised against residues 101–218; Sigma-Aldrich, Prestige Antibodies); anti-PDIA3 NBP-84797 (rabbit polyclonal, raised against residues 246–377; Novus Biologicals, Littleton, CO, USA), and anti-PDIA3 ABE1032 (rabbit polyclonal, raised against whole human recombinant PDIA3; Merck). Anti-human PDA1 antibody H-160 (rabbit polyclonal, raised against residues 211–370) was obtained from Santa Cruz. Anti-polyhistidine antibody A7058 (mouse monoclonal, peroxidase conjugate) was obtained from Sigma-Aldrich. Anti-Nus-Tag antibody (mouse monoclonal, with high affinity for NusA protein for detection of fusion proteins containing the Nus-Tag expressed with the pET-41.1 vector) was obtained from Novagen, EMD Chemicals, Merck. Second anti-mouse IgG (A3562), anti-rabbit IgG (A3687), and anti-goat IgG (A7650) antibodies, all conjugated with alkaline phosphatase, were obtained from Sigma-Aldrich. Rabbit anti-mouse IgG, conjugated with peroxidase (Cat. No. 315-035-003) was obtained from Jackson ImmunoResearch, West Grove, PA, USA.

### Immunoblotting

Immunoblotting was performed essentially as described before (Knoller et al., 1991), with a number of modifications. The blocking buffer consisted of NaCl (136 mM), Tris-HCl (25 mM), and KCl (2.68 mM), pH 7.5, supplemented 3 g% of bovine serum albumin (A4053, 96%, Sigma-Aldrich) and 0.1% (v/v) Triton X-100. A Pierce G2 Fast Blotter semi-dry transfer apparatus and 1-Step Transfer Buffer (Prod. No. 84731) were used, following the manufacturer's instructions (Thermo Fisher Scientific). Second antibodies were alkaline phosphatase conjugates and bands were detected by direct color development, using SIGMAFAST BCIP-NBT reagent (B5655, Sigma-Aldrich).

### Protein—protein binding assay

This assay was used to assess the binding of NusA-Nox2 fusion proteins to p67^*phox*^. p67^*phox*^ was diluted in phosphate buffered saline (PBS) (consisting of 137 mM NaCl, 2.7 mM KCl, 4.3 mM Na_2_PO_4_, and 1.4 mM KH_2_PO_4_, pH 7.3) to a concentration of 0.5 μM. 200 μl amounts were added to the wells of a 96-well plate (Immulon 4 HBX ultra-high binding flat bottom polystyrene, Cat. No. 3855, Thermo Labsystems, Helsinki, Finland). p67^*phox*^ was allowed to attach to the wells for 16–18 h at 4°C, the well contents being mixed on an orbital shaker. Unattached protein was removed by repeated washing with PBS supplemented with 0.1% v/v Tween 20 (Sigma-Aldrich), using a Wellwash Versa microplate washer (Thermo Fisher Scientific). Next, the surface-attached p67^*phox*^ was exposed to 200 μl/well of NusA-Nox2 or NusA, diluted to a concentration of 0.5 μM in PBS supplemented with 0.1% Tween 20 and 1% w/v casein, sodium salt (Sigma-Aldrich), for 1 h at room temperature, the well contents being mixed on an orbital shaker. After repeated washing, we added 200 μl/well anti-NusA antibody, diluted 1/2500 in in PBS supplemented with 0.1% Tween 20 and 1% w/v casein, for 1 h at room temperature with shaking. This was followed, after further washing, by 200 μl/well of peroxidase conjugated anti-mouse IgG, diluted 1/2500 in PBS supplemented with 0.1% Tween 20 and 1% w/v casein, and incubation for 1 h at room temperature, with shaking. After washing off the second antibody, bound peroxidase was quantified by adding 200 μl/well of tetramethyl benzidine + substrate chromogen reagent (DakoCytomation, Glostrup, Denmark) and measuring the increase in absorbance at 650 nm over time, for 10 min (Dahan and Pick, manuscript in preparation). Absorbance was measured in a Spectramax 340 microplate reader, in the kinetic mode (Molecular Devices, Sunnyvale, CA, USA) fitted with SoftMax Pro 5.2 software. Results were expressed as mAbs at 650 nm per min.

### Peptide—protein binding assay

This assay was used to assess the binding of p67^*phox*^, in solution, to surface-attached synthetic Nox2 peptides. The procedure is a modified version of that described before (Morozov et al., 1998; Dahan et al., 2002). A brief description of the procedure is presented here. Synthetic Nox2 peptides, with a biotin tag at either the N- or C-terminus, were diluted to a concentration of 1 μM in PBS supplemented with 1% casein. 200 μl/well volumes were added to streptavidin-coated 96 well plates (BioBind Assembly, streptavidin-coated, Cat. No. 95029263, Thermo Fisher Scientific) and the plates incubated for 1 h at room temperature, the well contents being mixed on an orbital shaker. After removing the unattached peptide by repeated washing with PBS supplemented with 1% v/v Tween 20, the wells were filled with 200 μl volumes of p67^*phox*^ at a concentration of 1.5 μM in PBS, supplemented with 1% casein, and the plates kept for 16–18 h at at 4°C with shaking on an orbital shaker. After removal of unattached p67^*phox*^ by repeated washing, 200 μl/well of a 1/3000 dilution of peroxidase-conjugated anti-polyhistidine antibody in PBS, supplemented with 0.1% Tween 20 and 1% w/v casein, were added and the plate incubated for 1 h at room temperature, with shaking. Bound peroxidase was quantified as described for the protein—protein binding assay.

### Measuring PDI reductase activity

The various methods of assaying PDI activity were recently reviewed (Watanabe et al., 2014). Preliminary experiments were performed using the turbidimetric assay based on the reduction of disulfides in oxidized insulin (Holmgren, 1979; Martinez-Galisteo et al., 1993) but this was abandoned when it was found not to be sufficiently sensitive for assessing the PDI activity of NusA-Nox2 fusion proteins. Consequently, we opted for a highly sensitive fluorescent method of measuring the disulfide reductase activity of PDI (Raturi and Mutus, 2007). The principle of this method is the use of DE-GSSG, in which two eosin molecules, covalently attached to glutathione disulfide (GSSG), exhibit fluorescent self quenching due to their proximity. On addition of PDI, in the presence of a minimal concentration of dithiothreitol (DTT), the disulfide bond in GSSG is reduced and the separation of the eosin molecules results in a ~70-fold increase in fluorescence. We first tested the feasibility of using this assay by measuring the activity of recombinant PDIA1 and PDIA3, following the assay conditions of Raturi and Mutus (2007). The assay mixture consisted of various concentrations of PDI in 1 ml volumes of 0.1 M potassium phosphate buffer, pH 7.0 supplemented with 2 mM EDTA, 150 nM DE-GSSG, and 5 μM DTT. Control mixtures consisted of PDI, in the absence of DTT, and of DTT, in the absence of PDI. The kinetics of fluorescence was initially measured in an FP-750 spectrofluorometer (JASCO Corporation, Tokyo, Japan), with excitation at 519 nm and emission, at 539 nm, in a stirred cuvette, for 15–30 min in the time course mode. The necessity to perform numerous measurements simultaneously and repeated measurements on the same sample led us to switch to the use of 96 well plates and a Gemini XPS microplate spectrofluorometer (Molecular Devices) fitted with SoftMax Pro 5.2 software. The assays were performed in Microfluor 1 Black flat bottom low background plates (Part 7605, Thermo Labsystems, Franklin, MA, USA). To each well were added, in a 200 μl volume, an assay mixture consisting of various concentrations of PDI in 0.1 M potassium phosphate buffer, pH 7.0 supplemented with 2 mM EDTA, 0.8 μM DE-GSSG, and 12.5 μM DTT. The kinetics of fluorescence was measured, with excitation at 519 nm and emission, at 545 nm, for 30 min, at 24°C, with six readings on the same well and mixing of the wells between readings. Results were expressed as *V*_max_ (milli relative fluorescence units per min), following selection of the linear segment of the increase in fluorescence curve.

### Ascertaining the absence of cysteines in the p67^*phox*^ mutant protein

The conversion of cysteines to serines in the p67^*phox*^ mutant was confirmed at the stage of constructing the mutant by DNA sequencing. We, nevertheless, ascertained the presence of cysteines in wild-type p67^*phox*^(1–212) and their absence in the mutant, by the binding or absence of binding of the thiol reagent monobromobimane (mBBR). mBBR is non-fluorescent when free and becomes fluorescent upon binding to thiol groups in proteins (Kosower and Kosower, 1995).

### Cell-free NADPH oxidase activation assays

Two variations of the cell-free NADPH oxidase assay were used. The canonical assay, known as the amphiphile- and p47^*phox*^-dependent assay, involves the participation of phagocyte membranes (as a source of cytochrome *b*_558_), p47^*phox*^, p67^*phox*^, Rac1 in the GTP-bound form, and an activating anionic amphiphile, such as arachidonate or lithium dodecyl sulfate (LiDS) (Bromberg and Pick, 1984, 1985). The second variation, known as the amphiphile- and p47^*phox*^-independent assay, involves the participation of phagocyte membranes, p67^*phox*^, and Rac1 and does not require an amphiphilic activator (Gorzalczany et al., 2000). Detailed descriptions of both methodologies have been published (Pick, 2014). In the vast majority of assays, NADPH oxidase activity was quantified by the production of superoxide (O^·−^_2_), measured by the reduction of oxidized cytochrome *c*. In some cases, when compounds, such as certain PDI inhibitors, interfered with cytochrome *c* reduction, we used NADPH consumption, as an alternative (Sha'ag, 1989). Results were expressed as turnover values (mol O^·−^_2_/s/mol cytochrome *b*_558_ heme).

### Effect of PDI inhibitors on cell-free NADPH oxidase avtivation

A number of agents described as PDI inhibitors were tested for an effect on cell-free NADPH oxidase activation. Based on the hypothesis that the target of such inhibitors is located in the phagocyte membrane and, specifically, in Nox2, the cell-free assays were designed in a manner to allow pre-incubation of the inhibitors with the phagocyte membranes for 5 min, at room temperature. This was followed by the addition of p47^*phox*^, p67^*phox*^, Rac1 and LiDS (in the canonical assay), or p67^*phox*^ and prenylated Rac1 (in the amphiphile- and p47^*phox*^-independent assay) and further incubation for 90 s or 5 min, respectively.

### Graph plotting

Plotting of graphs and kinetic analyses were executed by using GraphPad Prism Version 6.05 (GraphPad Software, San Diego, CA, USA).

## Results

### Exchanging the CGC TRIAD in Nox2 peptide 24 with CGHC or CGPC conserves but does not augment the enhancement of p67^*phox*^ binding

**Figure 1:**
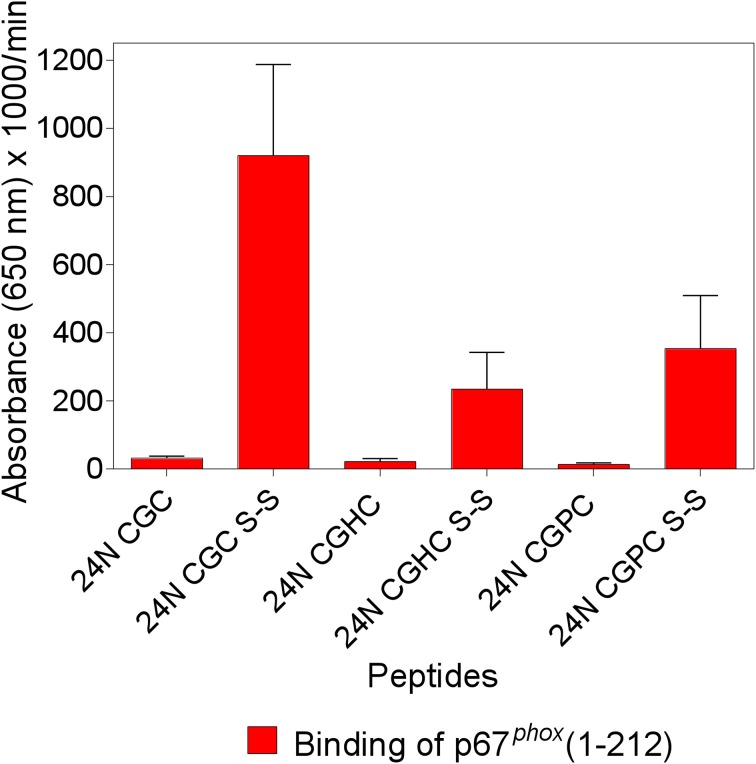
**The enhanced binding of p67^*phox*^ to Nox2 peptides with an intramolecular disulfide bond is maximal when the cysteines are separated by a single glycine**. The binding of p67^*phox*^(1–212), in solution, to surface-attached native and modified Nox2 peptide 24 (357–371), with an N-terminal biotin tag, was measured. The native peptide comprises the ^369^CysGlyCys^371^ triad at the C-terminus, in the dithiol form (labeled 24N CGC), and a disulfide form was synthesized, with an intramolecular disulfide bond between cysteines C369 and C371 (labeled 24N CGC S-S). Two more peptides were synthesized in which the CGC triad was replaced with CysGlyHisCys (labeled 24N CGHC) or CysGlyProCys (labeled 24N CGPC), to mimic the catalytic sites of PDI and thioredoxin, respectively. These two peptides were also synthesized in the dithiol form and with an intramolecular disulfide bond between cysteines 369 and 372. The methodology is described in the Materials and Methods Section. Results represent means ± SEM of three experiments.

We found that two synthetic 15-mer peptides, derived from the DHR of Nox2, which share a CGC triad at either the C- or N-terminus (designated peptides 24 and 28, respectively) bind full-length (1–526) and truncated (1–212) p67^*phox*^ with low affinity and the introduction of an intramolecular disulfide bond linking cysteines 369 and 371 leads to a marked increase in the binding of p67^*phox*^. Based on the resemblance of the CXC motif with the CXXC motif characteristic of the catalytic motifs of PDI and thioredoxin, we synthesized analogs of Nox2 peptide 24 (IVGDWTEGLFNACGC) in which the CGC sequence was replaced by either CGHC (the catalytic motif of PDI) or CGPC (the catalytic motif of thioredoxin). Both peptide analogs were prepared in the reduced and the disulfide forms and examined for the ability to bind p67^*phox*^, in comparison with the unmodified peptide 24. As apparent in Figure 1, the introduction of a disulfide bond in both modified peptides (CGHC and CGPC) caused a significant increase in p67^*phox*^ binding but the levels of binding were inferior to those measured with the disulfide form of the original peptide 24. Identical results were obtained when peptide 24 and the modified peptides, all in the reduced form, were subjected to oxidation by 1 mM H_2_O_2_, a procedure found to lead to the formation of intramolecular disulfide bonds between vicinal cysteines. These results are in good agreement with the findings of Woycechowsky and Raines (2003), showing that a CGC tripeptide, with an amidated C-terminus, is a good functional mimic of PDI.

### Alkylation or oxidation of thiols in p67^*phox*^ interferes with binding to Nox2 peptides containing the CGC triad

**Figure 2:**
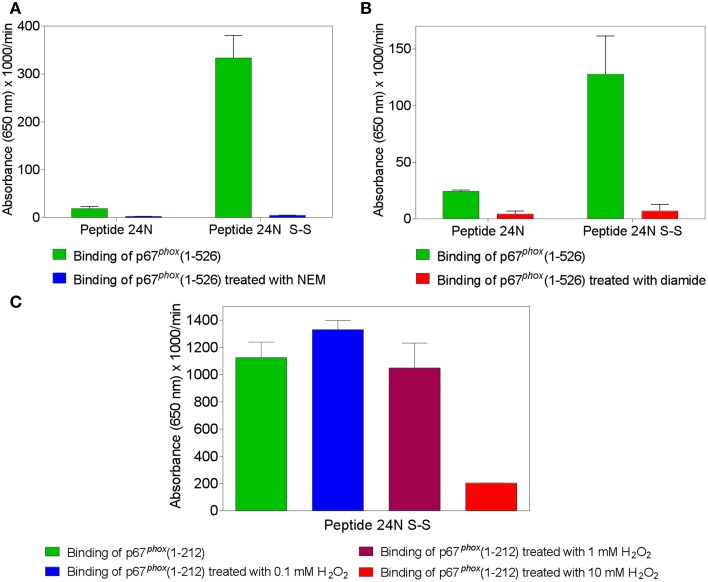
**Treatment of p67^*phox*^ with *N*-ethylmaleimide (NEM), diamide, or H_2_O_2_ reverses the enhanced binding to Nox2 peptides containing an intramolecular disulfide bond**. p67^*phox*^(1–526) (1.5 μM) was incubated with NEM (0.1 mM) **(A)** or diamide (5 mM) **(B)**, for 1 h at room temperature. p67^*phox*^(1–212) (1.5 μM) was exposed to H_2_O_2_ (0.1, 1, or 10 mM) for 2 h at 4°C **(C)**. Control preparations were supplemented with equal volumes of PBS for the same time interval. The binding of treated and untreated p67^*phox*^, in solution, to surface-attached Nox2 peptide 24 (357–371), with an N-terminal biotin tag, was measured. Binding to peptide 24 in the dithiol form (labeled 24N) and/or only (in the case of exposure to H_2_O_2_) to the peptide with an intramolecular disulfide bond between cysteines C369 and C371 (labeled 24N S-S) was assessed. Results shown in all panels represent means ± SEM of three experiments.

The findings that binding of p67^*phox*^ to Nox2 peptides containing the CGC triad was abolished when either C369 or C371 were exchanged to arginine or serine, and that Nox4 peptides, corresponding by alignment to Nox2 peptides 24 and 28, did not bind p67^*phox*^, as well as the clinical correlate showing that a C369 to R mutation in Nox2 causes the X91^+^ form of CGD, with impaired translocation of cytosolic components, represent strong indicators for disulfide bonds being established between Nox2 and p67^*phox*^ cysteines. To test this hypothesis directly, we treated p67^*phox*^ with the thiol alkylating agent NEM or the thiol oxidants diamide and H_2_O_2_ and measured binding to peptide 24 in the native and disulfide form. We found that pretreatment of p67^*phox*^ with either NEM (Figure 2A) or diamide (Figure 2B) totally abolished binding to Nox2 peptide 24 in the disulfide form. Both reagents also suppressed the low affinity binding to the native form of the peptide. These findings support the hypothesis that some form of thiol—disulfide exchange is involved in the binding of p67^*phox*^ to Nox2. Treatment of p67^*phox*^ with H_2_O_2_ also markedly reduced the binding of p67^*phox*^ to peptide 24 in the disulfide form but a surprisingly high concentration oh H_2_O_2_(10 mM) was required for the effect to take place (Figure 2C).

### Construction of recombinant fusion proteins comprising segments of the DHR of Nox2

**Figure 3:**
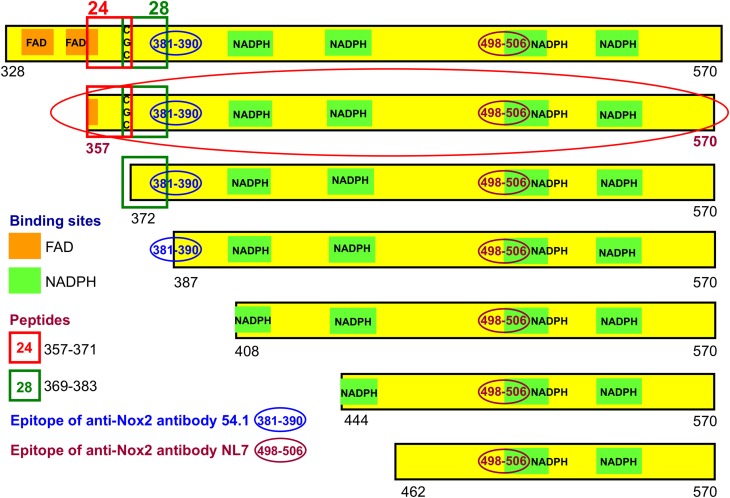
**Recombinant NusA-Nox2 proteins used in the present work**. A number of NusA-Nox2 fusion proteins, consisting of NusA and segments of the dehydrogenase region of Nox2, were constructed (see Materials and Methods and Supplementary Material). The figure shows a schematic representation of the serial N-terminal truncations of the Nox2 part of the fusion proteins. It also illustrates the location of peptides 24 (357–371) and 28 (369–383); the position of the FAD- and NADPH-binding sites, and the epitopes of the two monoclonal anti-Nox2 antibodies used in the present work. The truncation most used (357–570) is marked by a red-colored ellipse.

We have generated a number of recombinant fusion proteins consisting of the *E. coli* protein NusA and parts of the DHR of Nox2, starting at residues 328, 357, 372, 387, 408, 444, and 462, all ending at the C-terminus of Nox2 (residue 570) (Figure 3). As a control protein, we expressed NusA not fused to Nox2. Most work was performed with the fusion protein NusA-Nox2(357–570), which was the longest form which was predominantly soluble. Since all fusion proteins and NusA, too, consisted of a mixture of polymers and homodimers, we purified the proteins by gel filtration and used the homodimer fraction in all experiments. As seen in Figure 3, fusion protein NusA-Nox2(357–570) comprised residues 357–383, which correspond to the overlapping peptides 24 and 28, and, thus, contained the CGC triad. It also contained all parts of the NADPH-binding site and part of the FAD-ribityl binding site. The five shorter truncated proteins lacked the CGC triad.

**Figure 4:**
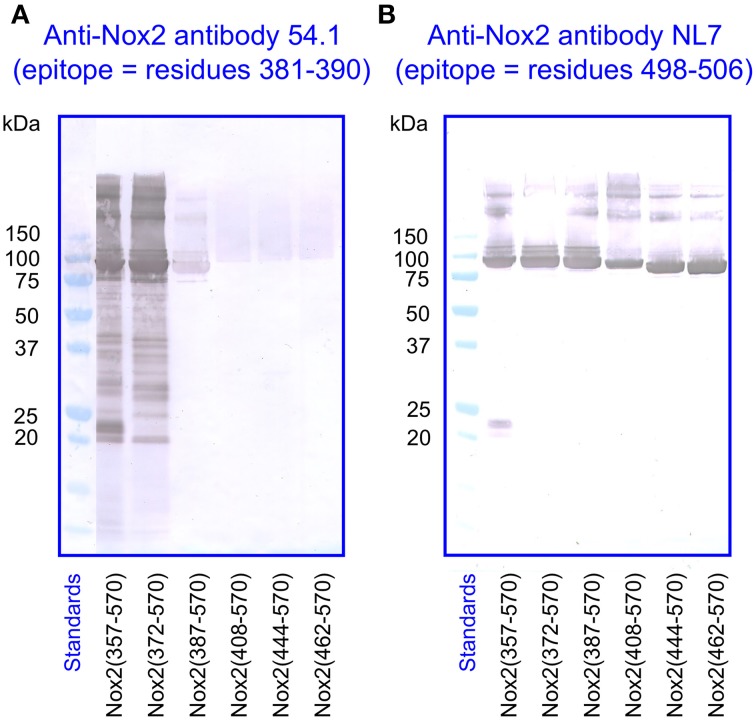
**Immunoblot of NusA-Nox2 fusion protein truncations using anti Nox2 monoclonal antibodies 54.1 and NL7. (A)** The figure illustrates the fact that antibody 54.1 (its epitope represented by Nox2 residues 381–390) recognizes Nox2 truncations 357–570 and 372–570, and reacts weakly with truncation 387–570. **(B)** Antibody NL7 (its epitope represented by Nox2 residues 498–506) recognizes all truncations, from 357–570 to 462–570.

For the immunologic characterization of the fusion proteins, we used two mouse monoclonal anti-human Nox2 antibodies. Antibody 54.1 reacted with Nox2 epitope 381-390 and antibody NL7 reacted with epitope 498–506. The locations of the two epitopes on the serially truncated Nox2 proteins are indicated in Figure 3. All truncated Nox2 proteins were subjected to immunoblot analysis. As apparent in Figure 4A, antibody 54.1 reacted with proteins NusA-Nox2(357–570) and (372–570) and weakly, with (387–570), in perfect correlation with the presence and absence of the relevant epitopes. Antibody NL7 reacted, as expected, with all the truncated proteins, in accordance with the presence of the relevant epitope on all truncations (Figure 4B). Thus, the structure of all NusA-Nox2 fusion proteins to be used in the present work, predicted by DNA sequencing, was confirmed by their immunologic characteristics.

### Binding of NusA-Nox2 fusion proteins to p67^*phox*^ is dependent on the presence of the CGC triad in Nox2

**Figure 5:**
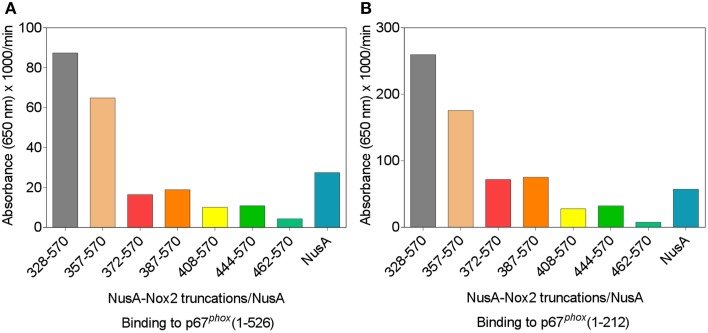
**p67^*phox*^(1–526) and p67^*phox*^(1–212) bind to NusA-Nox2(328–570) and NusA-Nox2(357–570) but not to shorter Nox2 truncations**. Binding of NusA-Nox2 fusion proteins and NusA, as a control, in the fluid phase, to surface-bound p67^*phox*^(1–526) **(A)** and p67^*phox*^(1–212) **(B)** was measured as described in the Materials and Methods Section. All proteins were purified by gel filtration on Superdex 200 and the homodimer-containing fractions were used in the binding studies. The results represent one characteristic experiment. The binding of p67^*phox*^ to NusA-Nox2(328–570) and NusA-Nox2(357–570) and the lack of binding to the five other truncations, which did not exceed the binding to NusA, was a constant feature of all experiments.

We used a protein—protein binding assay to measure the ability of the various NusA-Nox2 truncations to bind to p67^*phox*^. NusA not fused to Nox2 segments was used as a negative control. In these experiments, the homodimeric forms of NusA-Nox2 and NusA, derived by purification by gel filtration, were used. As seen in Figures 5A,B, only NusA-Nox2(328–570) and (357–570) exhibited significant binding to p67^*phox*^. The shorter truncations bound to p67^*phox*^ to an extent similar to that of unfused NusA. These results demonstrate the need for the presence of the CGC triad in Nox2 for binding to p67^*phox*^ since only NusA-Nox2(328–570) and (357–570) comprise the CGC triad (see Figure 3). Binding to p67^*phox*^(1–212) exceeded that to p67^*phox*^(1–526), in accordance with data indicating that a conformational change in p67^*phox*^(1–526) is required for optimal binding (Dang et al., 2002; Federman Gross et al., 2012).

### The NusA-Nox2 fusion protein exhibits PDI reductase activity

The availability of a soluble and well-characterized fusion protein comprising a major part of the DHR of Nox2 offered the opportunity to test it for the presence of enzymatic PDI activity. We first used the insulin reduction assay (Holmgren, 1979), with both the rate of aggregation and the lag time serving as kinetic parameters (Martinez-Galisteo et al., 1993). For standardizing the method, we assayed recombinant PDIA1 and PDIA3 and its sensitivity was found sufficient for measuring the activity of both PDIs, down to a concentration of 10 nM. We, thus, attempted to apply the insulin method for assessing the reductase activity of NusA-Nox2(357–570), up to a concentration of 5 μM, but no activity could be detected.

**Figure 6:**
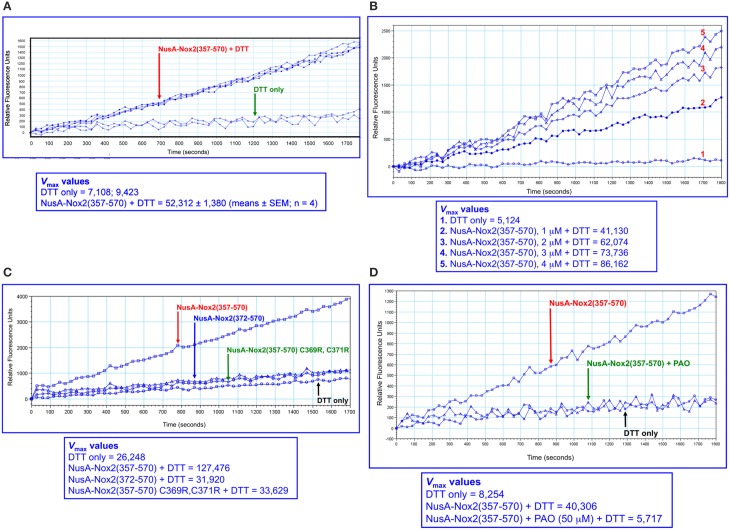
**PDI reductase activity of NusA-Nox2(357–570). (A)** Recombinant NusA-Nox2(357–570) (2 μM) was assayed for disulfide reductase activity on dieosin glutathione disulfide (DE-GSSG), in the presence of DTT, as described by Raturi and Mutus (2007) and detailed in the Materials and Methods Section. Briefly, the reaction mixtures contained 800 nM DE-GSSG and 12.5 μM DTT and the kinetics of the increase in fluorescence were followed for 30 min, using an excitation wavelength of 519 nm and an emission wavelength of 545 nm. Results are expressed as *V*_max_ (milli relative fluorescence units per min). **(B)** The dose dependence of the PDI reductase activity of NusA-Nox2(357–570) was assayed on DE-GSSG in the presence of DTT, as described in **(A)**. The concentration of NusA-Nox2(357–570) was varied from 1 to 4 μM. The results of one characteristic experiment are illustrated. **(C)** The absence of the ^369^CysGlyCys^371^ triad in NusA-Nox2(372–570) or mutating Cys 369 and Cys 371 to Arg in NusA-Nox2(357–570) eliminates PDI reductase activity. NusA-Nox2(357–570), NusA-Nox2(372–570), and NusA-Nox2(357–570) C369R, C371R (all, at a concentration of 2 μM) were assayed for disulfide reductase activity on DE-GSSG in the presence of DTT, as described in **(A)**. The results of one characteristic experiment are illustrated. **(D)** The PDI inhibitor phenylarsine oxide (PAO) interferes with the PDI activity of NusA-Nox2(357–570). Recombinant NusA-Nox2(357–570) (2 μM) was assayed for disulfide reductase activity on DE-GSSG in the presence of DTT, in the absence and presence of 50 μM PAO, as described in **(A)**. The results of one characteristic experiment are illustrated.

We next turned to using a higher sensitivity assay, in which the DTT-dependent disulfide reductase activity is measured by the relief of fluorescent self quenching of the PDI pseudo-substrate DE-GSSG, as described by Raturi and Mutus (2007). As apparent in Figure 6A, NusA-Nox(357–570), at a concentration of 2 μM, exhibited PDI activity in marked excess over that measured with DTT alone. The reductase activity showed clear dose dependency (Figure 6B). Since the only catalytic motif present in Nox2 that resembles the canonical motif of PDI (CGHC) is the CGC triad, we looked for a correlation between the presence of the CGC triad and activity. Such correlation was indeed found, as shown by the facts that NusA-Nox2(372–570), which lacks the CGC triad, as well as NusA-Nox2(357–570) in which C369 and C371 were mutated to arginine, exhibited an almost complete loss of PDI activity (Figure 6C). Further proof for the PDI activity expressed by NusA-Nox2 is shown by the fact that the PDI inhibitor, PAO (Gallina et al., 2002) abolished the DTT-dependent disulfide reductase activity at a concentration of 50 μM (Figure 6D). DTT could not be replaced by NADPH in the DE-GSSG reduction assay, when tested up to a concentration of 125 μM. No PDI activity was exhibited by the isolated Nox2 peptides 24(357–371) and 28(369–383), which contain the CGC triad, when assayed by DE-GSSG reduction over a wide concentration range.

**Figure 7:**
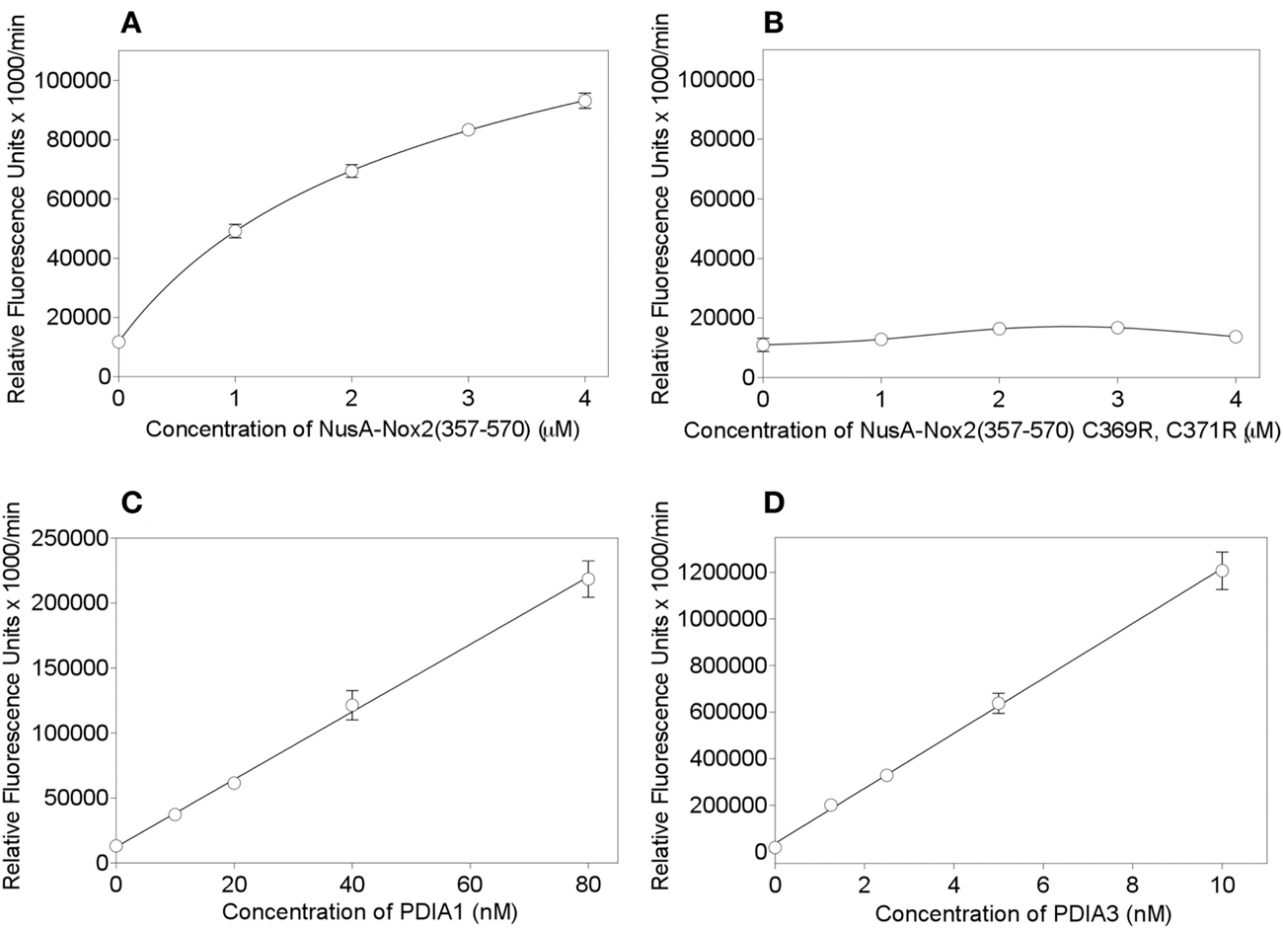
**PDI reductase activities of NusA-Nox2(357–570) compared to those of the NusA-Nox2(357-570) C369R, C371R mutant and to PDIA1 and PDIA3**. **(A)** PDI reductase activity of recombinant NusA-Nox2(357–570) was assayed in a concentration range of 1–4 μM and plotted as non-linear regression (one site binding equation). **(B)** PDI reductase activity of recombinant NusA-Nox2(357–570) C369R, C371R mutant was assayed in a concentration range of 1–4 μM and plotted as a cubic spline curve. **(C)** PDI reductase activity of recombinant PDIA1 was assayed in a concentration range of 10–80 nM and plotted as linear regression. **(D)** PDI reductase activity of recombinant PDIA3 was assayed in a concentration range of 1–10 nM and plotted as linear regression. The assays were performed as described by Raturi and Mutus (2007) and detailed in the Materials and Methods Section. Results represent means ± SEM of 9 (NusA-Nox2(357–570), 4 (NusA-Npx2(357–570) C369R, C371R), 4 (PDIA1), and 3 (PDIA3) experiments.

In order to place Nus-Nox2 in the context of canonical PDIs, we performed dose-response reductase assays with NusA-Nox2(357–570) in comparison to recombinant PDIA1 and PDIA3. The concentration ranges for each protein were chosen to assure a linear increase in fluorescence within a sufficiently long time interval to allow reliable* V*_max_ calculations. As seen in Figure 7A, the activity of NusA-Nox2(357–570) (1–4 μM) best fitted a one site binding hyperbola. The NusA-Nox2(357–570) C369R, C371R mutant was found to lack reductase activity at concentrations of 1–4 μM and, thus, no dose-response curve could be fitted (Figure 7B). The activities of both PDIA1 (10–80 nM) and PDIA3 (1.25–10 nM) generated linear regression curves (Figures 7C,D). By using extrapolation of *V*_max_ values obtained with a concentration of 10 nM PDIA1 (37,303 ± 1279) and 10 nM PDIA3 (1,206,911 ± 81,022) to the *V*_max_ measured with 1 μM NusA-Nox2(357–570) (49,164 ± 2271), we calculated that PDIA1 was about 75 times and PDIA3, 2400 times more active than NusA-Nox2(357–570). It is of possible interest that, using this assay, the reductase activity of PDIA3 was about 30 times higher than that of PDIA1.

### Nox2 and PDIA3 share small regions of sequence homology

**Figure 8:**
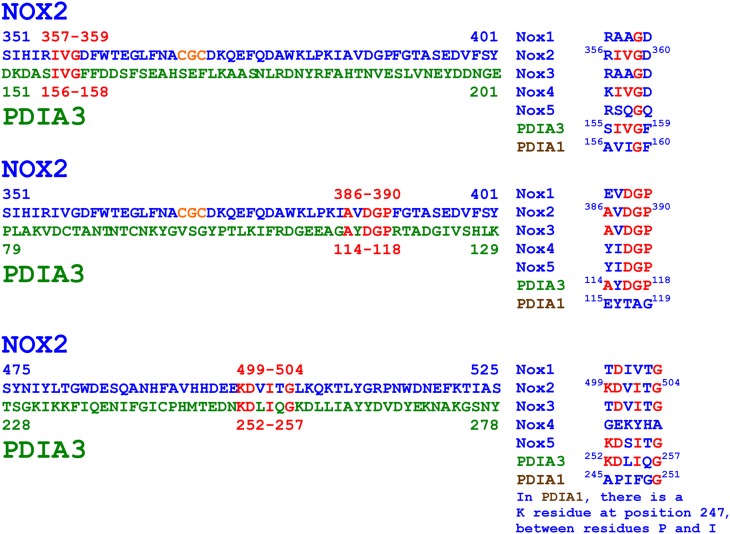
**Amino acid sequence similarities between the dehydrogenase region of Nox2 and PDIA3**. The figure illustrates three small regions (3–6 residues) exhibiting partial sequence identities between Nox2 and PDIA3. The representation of the motifs in non-phagocytic Noxes (Noxes 1, 3, 4, and 5) is also shown. Monoclonal anti-Nox2 antibody 54.1 recognizes Nox2 residues ^381^KLPKIAVDGP^390^ and might recognize PDIA3 residues ^114^AYDGP^118^ (see Figure 9A). Monoclonal anti-Nox2 antibody NL7 recognizes Nox2 residues ^498^EKDVITGLK^506^ and might recognize PDIA3 residues ^252^KDLIQG^257^ (see Figure 9B). Polyclonal anti-PDIA3 antibody H-220 was raised against PDIA3 residues 108–207 and could potentially recognize PDIA3 ^114^AYDGP^118^ and ^156^IVG^158^ and Nox2 residues ^386^AVDGP^390^ and ^357^IVG^359^, respectively (see Figure 10A). The regions of Nox2/PDIA3 homology do not exist in the sequence of PDIA1, in good agreement with the fact that the two anti-Nox2 antibodies do not recognize PDIA1 (see Figures 9A,B) and a polyclonal anti-PDIA1 antibody does not recognize Nox2 (see Figure 10B). Identical residues in different proteins are in red font; the CGC triad in Nox2 is in orange font.

In the course of the generation of a mouse monoclonal anti-Nox2 antibody (54.1), it was found that the antibody reacted with a protein known as GRp58 or ERp57, which is identical to the PDIA3 member of the PDI family (Baniulis et al., 2005). The authors noted that the cross-reaction was explained by Nox2 and PDIA3 sharing a five-residues motif (AVDGP, in the DHR of Nox2, and AYDGP, in PDIA3). We noticed that yet another mouse monoclonal antibody to Nox2 (NL7) reacted with an epitope in the DHR of Nox2 (KDVITG) which shares four residues with sequence KDLIQG, in PDIA3. Finally, yet another minor, three-residue identity between the DHR of Nox2 and PDIA3 (IVG), was noticed (for all similarities, see Figure 8). The above Nox2 sequences exhibit no similarity with PDIA1.

The residues in the Nox2 sequence AVDGP shared with PDIA3 are also present in Nox3; the shared residues in Nox2 sequence KDVITG are also present in Nox5, and shared residues IVG are also present in Nox4. However, as noted in the Introduction, the CGC triad is specific for Nox2. The AYDGP sequence in PDIA3 is located in the catalytic domain *a*, C-terminal to the CGHC motif; and the IVG and KDLIQG sequences are located in domains *b* and *b*′, respectively (Coe and Michalak, 2010; Kozlov et al., 2010).

### Immunoblot analysis confirms Nox2/PDIA3 sequence similarities

**Figure 9:**
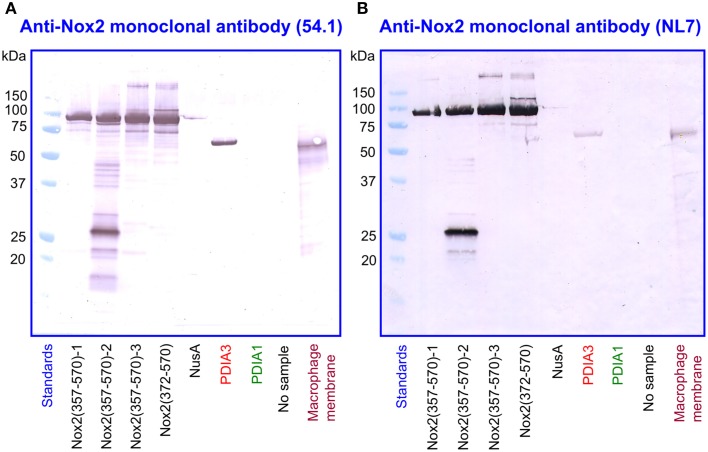
**Two monoclonal anti-Nox2 antibodies react with PDIA3 but not with PDIA1 by immunoblotting. (A)** Monoclonal anti-Nox2 antibody 54.1 (epitope, residues 381–390) reacts with recombinant NusA-Nox2(357–570) and NusA-Nox2(372–570) but not with NusA. It reacts strongly with recombinant PDIA3 but not with recombinant PDIA1. It recognizes a protein of about 54–58 kDa (diffuse band) in the guinea pig macrophage membrane, corresponding to Nox2 (Knoller et al., 1991). **(B)** Monoclonal anti-Nox2 antibody NL7 (epitope, residues 498–506) reacts with recombinant NusA-Nox2(357–570) and NusA-Nox2(372–570) but not with NusA. It reacts moderately with recombinant PDIA3 but not with recombinant PDIA1. It recognizes a protein of about 54–58 kDa (diffuse band) in the guinea pig macrophage membrane, corresponding to Nox2. Nox2(357–570) numbered 1, 2, and 3 represent three batches of NusA-Nox2(357–570).

We subjected preparations of NusA-Nox2(357–570) and (372–570), NusA, recombinant PDI3 and PDIA1, and macrophage membranes to immunoblotting with the two anti-Nox2 antibodies 54.1 and NL7. As apparent in Figures 9A,B, anti-Nox2 antibodies 54.1 and NL7 reacted with PDIA3 but not with PDIA1. The reactivity of antibody 54.1 with PDIA3 was more pronounced than that of antibody NL7. As expected, and as shown in Figure 4, both antibodies reacted strongly with NusA-Nox2(357–570) and (372–570) and detected a broader and more diffuse band of about 54–58 kDa in macrophage membranes, corresponding to the characteristics of guinea pig Nox2 (Knoller et al., 1991). A polyclonal goat anti-Nox2 antibody (C-15), raised against a peptide in the C-terminal region of Nox2, which reacted with NusA-Nox2(357-570), did not react with PDIA3 (result not shown, see Table 1). We suggest that the ability of the two anti-Nox2 antibodies to react with PDIA3 is due to the similarity of the Nox2 epitopes recognized by antibodies 54.1 and NL7 with sequences AYDGP and KDLIQG in PDIA3, respectively (see Figure 8).

**Table 1:** **Cross-reactivity of some anti-Nox2 antibodies with PDIA3 and of some anti-PDIA3 antibodies with Nox2**.

**Protein**	**Antibodies**							
	**Anti-Nox2 54.1**	**Anti-Nox2 NL7**	**Anti-Nox2 C-15**	**Anti-PDIA3 H-220**	**Anti-PDIA3 HPA003230**	**Anti-PDIA3 NBP1-84797**	**Anti-PDIA3 ABE1032**	**Anti-PDIA1 H-160**
	**Mouse monoclonal**	**Mouse monoclonal**	**Goat polyclonal**	**Rabbit polyclonal**	**Rabbit polyclonal**	**Rabbit polyclonal**	**Rabbit polyclonal**	**Rabbit polyclonal**
NusA-Nox2 (357–570)	+	+	+	+	−	−	−	−
NusA-Nox2 (372–570)	+	+	ND^a^	−	−	ND^a^	−	ND^a^
PDIA3 (recombinant)	+	+	−	+	+	+	+	+/−^b^
PDIA3 (in macrophage membrane)	+	+	ND^a^	+	+	+	+	+^c^
PDIA1 (recombinant)	−	−	−	−	−	−	+/−^b^	+

a *ND, not determined*.

b *Weak reaction in comparison to that with the specific antigen*.

c *PDIA1 was detected; detection of PDIA3 could not be established with certainty because of similarity in size with PDIA1*.

**Figure 10:**
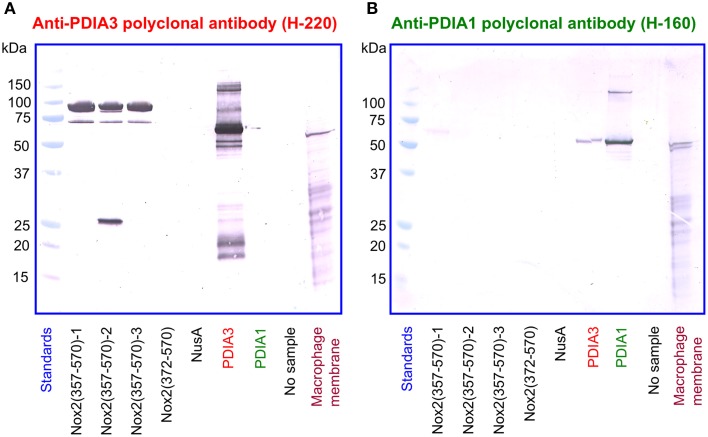
**A polyclonal anti-PDIA3 antibody but not a polyclonal anti-PDIA1 antibody reacts with Nox2 by immunoblotting**. **(A)** Polyclonal anti-PDIA3 antibody H-220 reacts with recombinant NusA-Nox2(357–570) but not with NusA-Nox2(372–570) and NusA. It reacts strongly with recombinant PDIA3 but not with recombinant PDIA1. It also recognizes a protein in the guinea pig macrophage membrane (sharp single band). **(B)** Polyclonal anti-PDIA1 antibody H-160 does not react with recombinant NusA-Nox2(357–570) and NusA-Nox2(372–570). It reacts with recombinant PDIA1 and weakly, with recombinant PDIA3. It also recognizes a protein in the guinea pig macrophage membrane (sharp double band). Nox2(357–570) numbered 1, 2, and 3 represent three batches of NusA-Nox2(357–570).

In a reciprocal series of experiments, we found that the polyclonal anti-PDIA3 antibody H-220 reacts strongly with NusA-Nox2(357–570) but not with Nusa-Nox2(372–570) and NusA (Figure 10A). Its specificity is proven by its ability to recognize recombinant PDIA3 but not PDIA1. The antibody does not seem to recognize Nox2 in macrophage membranes in spite of the high level of homology of human and guinea pig Nox2. It, however, detects a sharp band in the macrophage membrane, of a size somewhat smaller than recombinant PDIA3 (see Discussion of this finding in the paragraph below). In contrast to anti-PDIA3, polyclonal anti-PDIA1 antibody does not react with NusA-Nox2(357–570) (Figure 10B). Its specificity is shown by it recognizing recombinant PDIA1, though there is some cross-reaction with PDIA3. Anti-PDIA1 also recognizes an antigen in the macrophage membrane (double band), possibly PDIA1. These findings are best explained by the likelihood that polyclonal anti-PDIA3 antibody H-220, raised against residues 108–207 of PDIA3, comprises antibodies against sequence AYDGP (residues 114–118), similar to sequence AVDGP (residues 386–390) in Nox2, and, possibly IVG (residues 156–158), identical to sequence IVG (residues 357–359) in Nox2. We have no explanation for the lack of reaction of anti-PDIA3 H-220 with truncation NusA-Nox2(372–570), unless the participation of epitope IVG [absent in NusA-Nox2(372–570)] is required.

**Figure 11:**
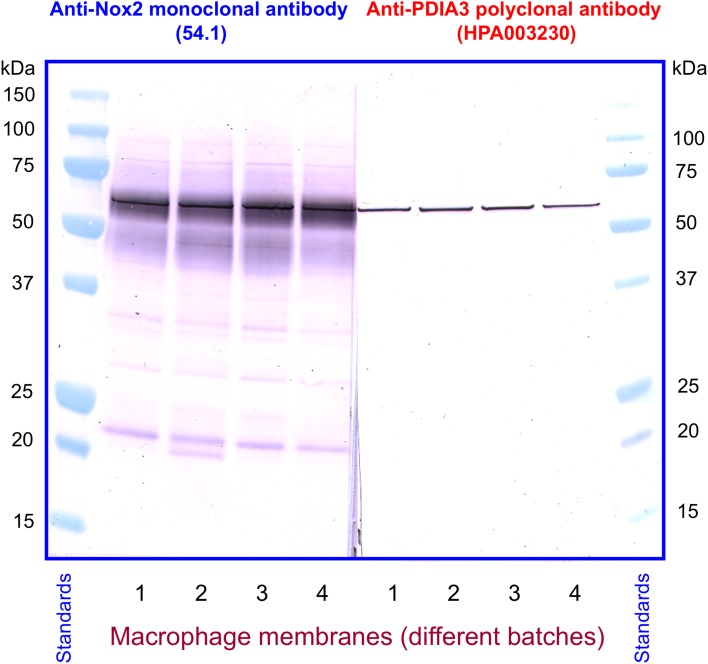
**Detection of PDIA3 in macrophage membranes by both anti-Nox2 and anti-PDIA3 antibodies**. Monoclonal anti-Nox2 antibody 54.1, which recognizes recombinant PDIA3 (see Figure 9A), is shown to detect in macrophage membranes an antigen most likely to be PDIA3. This appears in four distinct membrane batches as a sharply defined band on the background of the more diffuse Nox2; the overlap is caused by the almost identical size of guinea pig Nox2 and PDIA3. A polyclonal anti-PDIA3 antibody (HPA003230), found not to cross-react with Nox2 (see Table 1), detects PDIA3 in macrophage membranes. The position and character of the bands detected by anti-PDIA3 strengthens the proposal that the sharp band detected by anti-Nox2 represents PDIA3.

We next investigated the identity of the sharp band detected by anti-PDIA3 H-220 in macrophage membranes. For this purpose, we immunoblotted four individual batches of macrophage membranes with monoclonal anti-Nox2 antibody 54.1, in parallel with four polyclonal anti-PDIA3 antibodies (H-220, HPA003230, NBP-84797, and ABE1032; the characteristics of the antibodies are described in Materials and Methods). With the exception of H-220, none of the anti-PDIA3 antibodies recognized recombinant NusA-Nox2(357–570) and none of the antibodies recognized Nox2 in macrophage membranes. However, all four anti-PDIA3 antibodies detected a protein of about 58 kDa in all batches of macrophage membranes in the shape of a sharply defined narrow band, indicating that PDIA3 is present in the membrane (see Figure 11, illustrating results obtained with anti-Nox2 antibody 54.1 and anti-PDIA3 antibody HPA003230). Monoclonal anti-Nox2 antibody 54.1 reacted with Nox2 in membranes (a diffuse band) but also with what appears to be PDIA3 present in the membrane, appearing as a narrow band on the background of the more diffuse Nox2, corresponding in size to the band detected by anti-PDIA3 antibody HPA003230. The presence of PDIA3 in macrophage membranes might offer the explanation for the inability of anti-PDIA3 antibody H-220 to recognize Nox2 in the membrane; this could be due to competition by membrane PDIA3, to which the antibody is likely to bind with higher affinity. All results obtained by immunoblotting are summarized in Table 1. The presence of PDIA3 (and PDIA1) in macrophage membranes, although expected in light of the ubiquity of PDIs even outside the endoplasmic reticulum (Turano et al., 2002), poses a methodological challenge when investigating the PDI-like function of Nox2 and emphasizes the advantage of working with recombinant Nox2.

### Mutating cysteines in p67^*phox*^(1–212) to serine prevents binding to a Nox2 DHR peptide containing the CGC triad

The data presented so far suggest that the CGC triad in the DHR of Nox2 serves as a pseudo-PDI catalytic motif leading to the binding of p67^*phox*^ to Nox2 via a thiol-disulfide exchange reaction. Since such a reaction must involve cysteine(s) in p67^*phox*^ and since oxidation or alkylation of cysteines in p67^*phox*^ abolished binding to CGC-containing Nox2 peptides (see Figure 2), we reasoned that mutating the cysteines in p67^*phox*^ should prevent binding to these peptides and also affect oxidase activation.

Full-length p67^*phox*^ contains nine cysteines and p67^*phox*^(1–212) contains four. Based on the knowledge that p67^*phox*^(1–212) binds to NusA-Nox2 and to CGC-containing Nox2 peptides and supports oxidase activation* in vitro* with an efficiency identical to that of the full-length protein, we mutated the four cysteines present in p67^*phox*^(1–212) (cysteines 40, 45, 121, and 165) to serines. The wild-type protein and the mutated protein were found by gel filtration to have a native molecular mass of 28.4 and 27.4 kDa, respectively, in good agreement with the theoretical value of 25.5 kDa. The only difference between the two proteins was an apparent increase in the hydrophobic character of the mutant protein (see Materials and Methods). We confirmed the absence of cysteines in the mutant protein by comparing the binding of the thiol probe mBBr (Kosower and Kosower, 1995) to the wild-type and mutant proteins. Wild-type protein indeed bound mBBr, whereas the mutant protein did not (results not shown).

**Figure 12:**
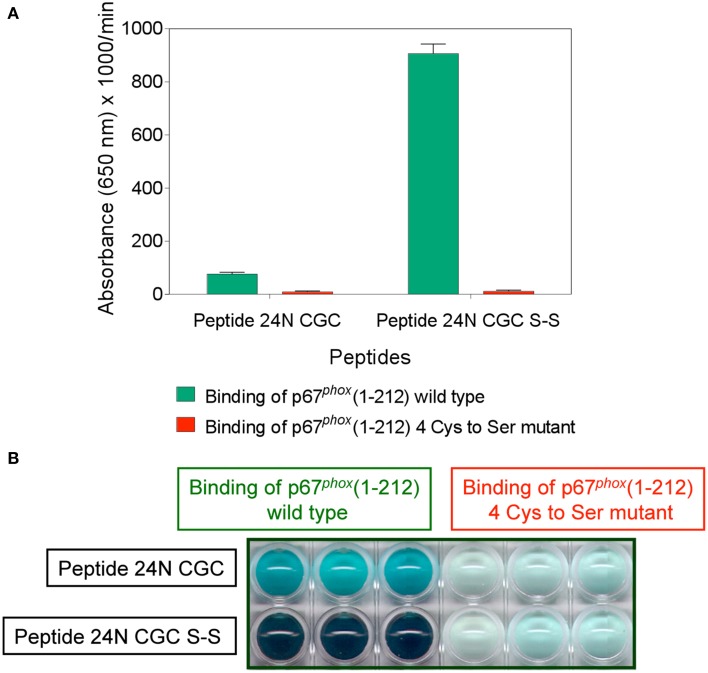
**Mutating all four cysteines in p67^*phox*^(1–212) eliminates its ability to bind to Nox2 peptide 24**. Binding of wild type p67^*phox*^(1–212) and p67^*phox*^ in which ^40^Cys, ^45^Cys, ^121^Cys, and ^165^Cys were mutated to Ser, in solution, to surface-attached Nox2 peptide 24 (357–371), with an N-terminal biotin tag, in the dithiol form (labeled 24N CGC), and with an intramolecular disulfide bond between cysteines C369 and C371 (labeled 24N CGC S-S), was assessed. The methodology is described in the Materials and Methods Section. **(A)** Binding of the mutant protein to both forms of the peptide is markedly reduced. Results represent means ± SEM of 3 experiments. **(B)** End point view of the wells of a 10 min kinetic experiment **(A)**. The depth of the blue-green color, representing oxidized TMB, is proportional with the binding of wild type and mutant p67^*phox*^ to the peptides. Results are those of a single representative experiment.

The binding of wild type and mutant p67^*phox*^ to Nox2 DHR peptide 24 comprising the CGC triad, in either the dithiol or disulfide form, was assessed by the peptide—protein binding assay. As apparent in Figures 12A,B, the wild type protein exhibited moderate binding to peptide 24 in the reduced form and much enhanced binding to the disulfide form. The mutant protein lacked binding ability to both forms of the peptide. The total absence of binding of the mutant protein to the disulfide form of peptide 24 is especially noteworthy.

### Mutating cysteines in p67^*phox*^(1–212) to serine interferes with NADPH oxidase activation

**Figure 13:**
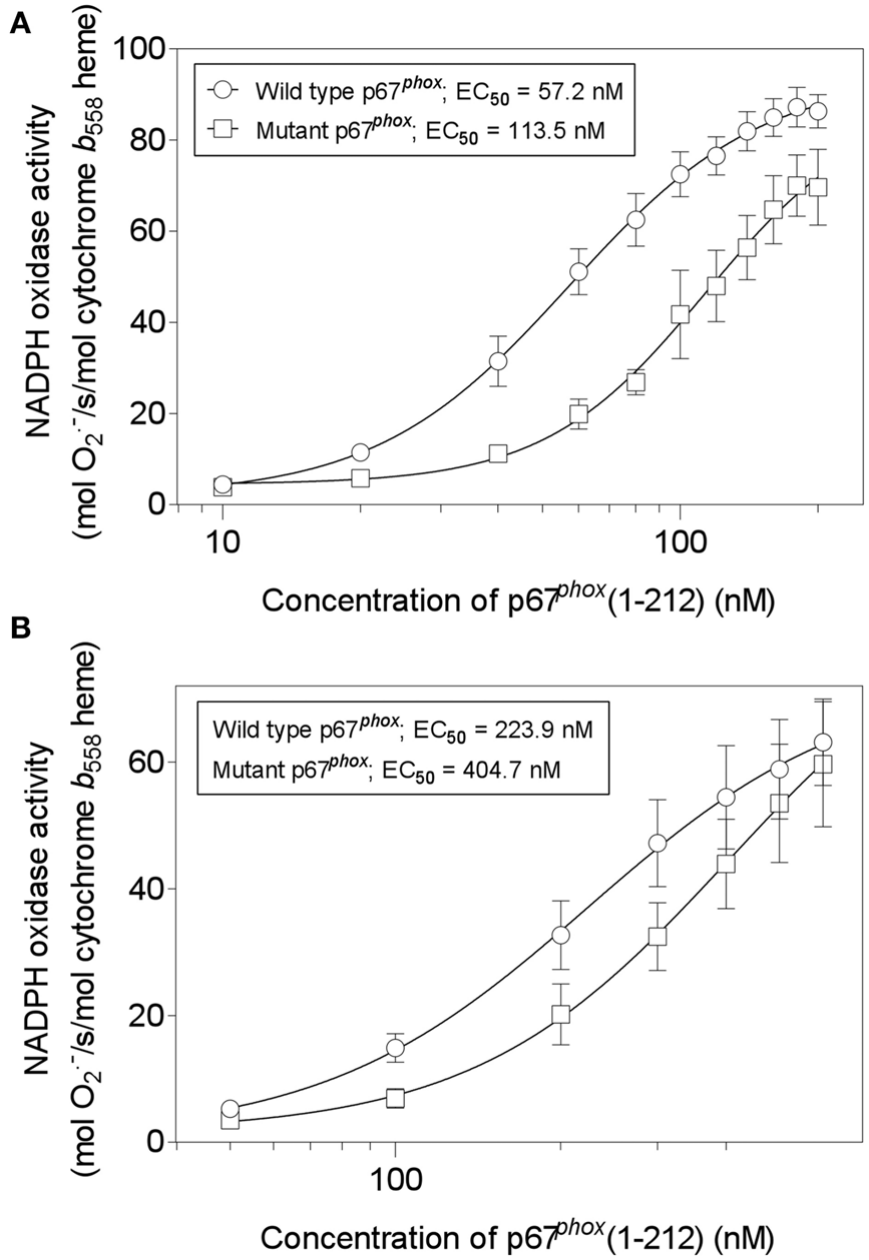
**Mutating all four cysteines in p67^*phox*^(1–212) impairs its ability to support NADPH oxidase activation in a cell-free system**. Wild-type p67^*phox*^(1–212) and p67^*phox*^ in which ^40^Cys, ^45^Cys, ^121^Cys, and ^165^Cys were mutated to Ser, were assayed for the ability to support NADPH oxidase activation in a cell-free system. Two forms of assay were used. **(A)** The first (amphiphile- and p47^*phox*^-dependent) consisted of macrophage membrane liposomes (5 nM cytochrome *b*_558_ heme), equal concentrations of p67^*phox*^ (wild-type or mutant), p47^*phox*^, and Rac1 Q61L, varying from 10 to 200 nM, and LiDS (120 μM). Activation proceeded for 90 s, followed by addition of 240 μM NADPH, to initiate O^·−^_2_ production. **(B)** The second (amphiphile- and p47^*phox*^-independent) consisted of macrophage membrane liposomes (5 nM cytochrome *b*_558_ heme) and equal concentrations of p67^*phox*^ (wild-type or mutant) and prenylated Rac1 Q61L, varying from 50 to 600 nM. Activation proceeded for 5 min, followed by addition of 240 μM NADPH, to initiate O^·−^_2_ production.

We next assayed wild-type and mutant p67^*phox*^(1–212) for their ability to support oxidase activation in the cell-free system. We compared the two proteins in the amphiphile- and p47^*phox*^-dependent and in the amphiphile- and p47^*phox*^-independent systems. As seen in Figures 13A,B, there was a two-fold increase in the EC_50_ of the mutant p67^*phox*^ in both assays, indicating that that the mutant protein has a significantly impaired ability to support oxidase activation. In accordance with our hypothesis, the mutant protein did not loose its activity completely, as expected from an effect of the stability of the assembled oxidase complex, as opposed to an effect on the primary interaction, which would result in a total loss of activity. Cysteine mutagenesis was applied in the past to p47^*phox*^ and shown to result in a paradoxical increase in the ability to support oxidase activation by an unknown mechanism (Babior, 2002).

### The majority of PDI inhibitors do not affect NADPH oxidase activation *in vitro*

It has been reported that two PDI inhibitors, bacitracin and scrambled RNAs, inhibit oxidase activation in an amphiphile-dependent cell-free system, consisting of neutrophil membranes and recombinant cytosolic components (Paes et al., 2011). As referred to in the Introduction, PAO and gliotoxin, two compounds binding to vicinal cysteines and, most likely, acting on C369 and C371 in Nox2, were reported to inhibit oxidase activation. PAO is considered to act as a PDI inhibitor (Gallina et al., 2002) and direct evidence for this was presented by Raturi et al. (2005) and also appears in Figure 6D. Direct proof for gliotoxin acting as a PDI inhibitor is lacking.

**Figure 14:**
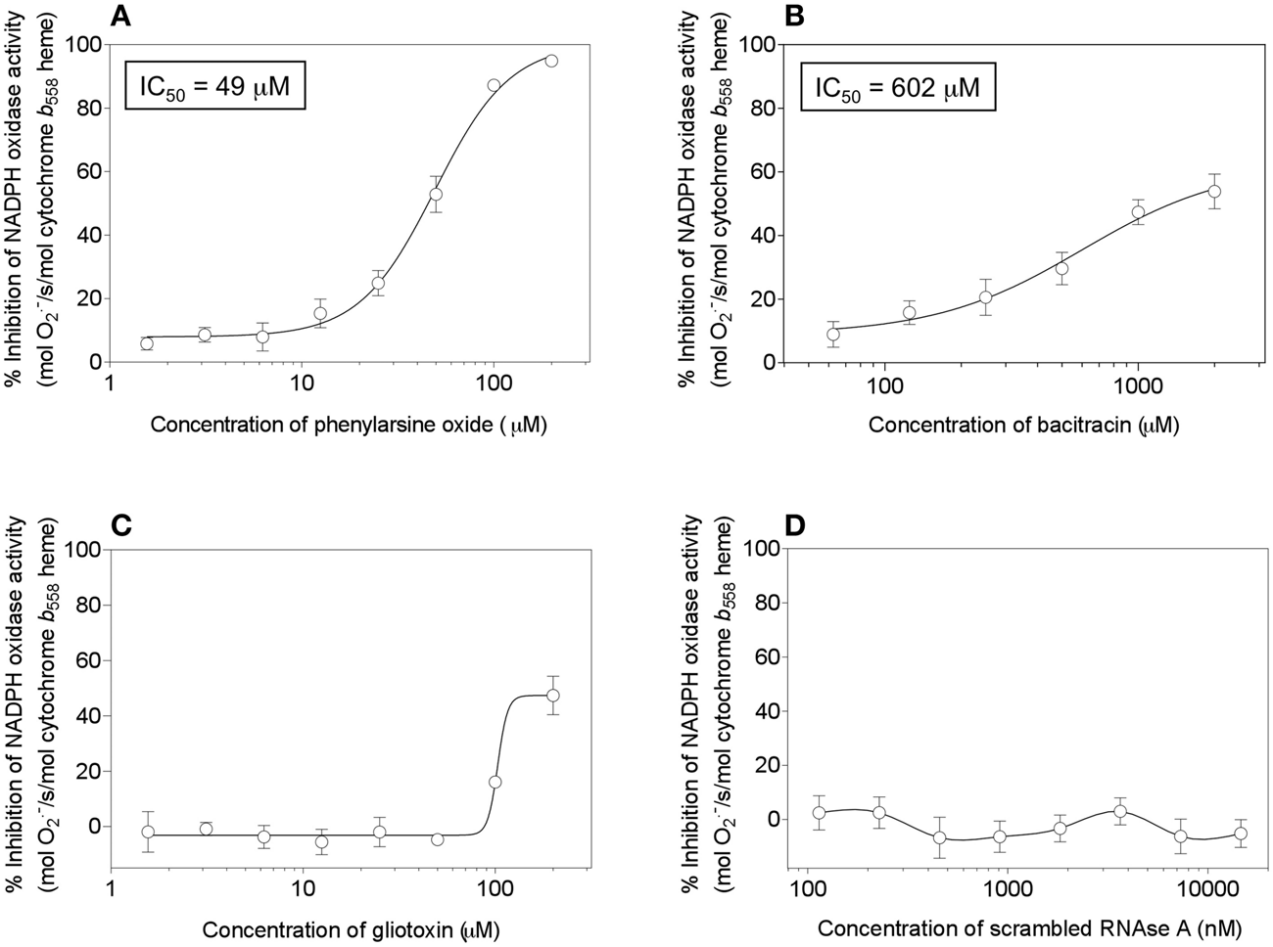
**Effect of PDI inhibitors on NADPH oxidase activation in a cell-free system**. Selected PDI inhibitors were tested for their ability to interfere with NADPH oxidase activation in an amphiphile- and p47^*phox*^-independent system. The reaction mixtures contained macrophage membrane liposomes (5 nM cytochrome *b*_558_ heme), p67^*phox*^(1–212) (300 nM), and prenylated Rac1 Q61L (300 nM). The membrane liposomes were preincubated with the PDI inhibitors for 5 min, followed by the addition of p67^*phox*^ and Rac1, incubation for 5 min in the absence of an amphiphilic activator, and the addition of 240 μM NADPH, to initiate O^·−^_2_ production. The effect of the following inhibitors is described in the figure: **(A)** phenylarsine oxide (1.56–200 μM); **(B)** bacitracin (62.5–2000 μM); **(C)** gliotoxin (1.56–200 μM), and **(D)** scrambled RNAse (0.114–14.6 μM). Results represent means ± SEM of 3 experiments, for each of the inhibitors tested. IC_50_ values are indicated for phenylarsine oxide and bacitracin but could not be determined for gliotoxin and scrambled RNAse, due to the absence of curves amenable to kinetic analysis.

We, thus, engaged in a systematic investigation of the effect of a number of compounds, reported to act as PDI inhibitors on a variety of targets and in various situations, on cell-free oxidase activation. The amphiphile- and p47^*phox*^-independent assay was used, in order to reduce the number of potential targets for the inhibitors. Since p47^*phox*^ was described to associate with exogenous PDI (Paes et al., 2011), we preferred to work in a system free of p47^*phox*^. In addition to PAO and gliotoxin, the following PDI inhibitors were tested: bacitracin (Dickerhof et al., 2011), scrambled RNAse (Essex and Li, 1999), 16F16 (Hoffstrom et al., 2010), rutin (Jasuja et al., 2012), and PACMA 31 (Xu et al., 2012). In all assays, the potential inhibitor was preincubated with the membrane component before the induction of oxidase complex assembly. As shown in Figure 14, significant inhibition of oxidase activation was caused only by PAO (Figure 14A), in accordance with earlier work (Le Cabec and Maridonneau-Parini, 1995; Doussiere et al., 1998). However, in preliminary experiments (results not shown), we found that PAO also inhibited oxidase activation supported by p67^*phox*^ with cysteines mutated to serines, a finding which raises questions about its mechanism of action. It was, indeed, reported that PAO also had an effect on the heme of Nox2 (Doussiere et al., 1999). Gliotoxin was found to be a poor inhibitor, when tested up to a concentration of 200 μM, and generated an atypical dose-response curve, a result which differed from the marked inhibitory effect reported in the past (Nishida et al., 2005). Bacitracin was also found to be a poor inhibitor, with an IC_50_of 602 μM and a maximal inhibition not exceeding 60%, and scrambled RNAse was ineffective, up to a concentration of 14.6 μM. Lack of an inhibitory effect was also found for 16F16 (up to 14.6 μM), rutin (up to 400 μM), and PACMA 31 (up to 500 μM).

These results do not counter our hypothesis that the DHR of Nox2 functions as a PDI. Nox2 possesses only one copy of a catalytic site resembling CGHC and lacks the equivalent of a substrate binding domain (*b*′). Bacitracin is unlikely to be an effective inhibitor, first because its target in PDI is the *b*′ domain and its linker to domain *a'*, both absent in Nox2, and second because commercial bacitracin consists predominantly of bacitracin A, of low inhibitory potency (Dickerhof et al., 2011). Isomerization by PDI also requires the *b*′ domain (Ellgaard and Ruddock, 2005) and, thus, an interaction between Nox2 and scrambled RNAse is unlikely. The mechanism of action of 16F16 and rutin are not fully understood but it is of interest that rutin was found to lack an inhibitory effect on PDIA3 (Jasuja et al., 2012). The lack of effect of PACMA 31 is also expected, considering the exquisite specificity of the compound for PDIA1 and, more specifically for cysteines in the catalytic motif in domain *a'*, in conjunction with additional non-cysteine residues (Xu et al., 2012).

## Discussion

Our results support a model of NADPH oxidase assembly and consequent activation in which the cytosolic component p67^*phox*^, most directly responsible for activation, establishes a disulfide bond with a cysteine belonging to the ^369^CysGlyCys^371^ triad in the DHR of Nox2. This interaction serves as a stabilizer of the oxidase complex and is, most likely, secondary to the primary binding of p67^*phox*^ to Nox2, which involves specific binding sites on both components.

Earlier findings that were suggestive of such a mechanism were: (a) p67^*phox*^ binds to Nox2 peptides sharing the CGC triad at either the C- or N-termini; (b) p67^*phox*^ does not bind to peptides when either C369 or C371 are replaced by arginine or serine; (c) The CGC triad is present only in Nox2, the most potent generator of superoxide among all members of the Nox family, and the one most dependent on regulation by cytosolic components, and (d) The introduction of a disulfide bond between C369 and C371 in Nox2 peptides greatly enhances binding of p67^*phox*^ and its reduction to the dithiol form abolishes the binding.

Work by other groups also supports our hypothesis. Thus, patients with a C369 to R mutation in Nox2 suffer from the X91^+^ form of CGD, with normal expression of Nox2 but impaired translocation of cytosolic components (Leusen et al., 2000; Debeurme et al., 2010). It was also reported that exposure of phagocyte membranes to H_2_O_2_ or of p67^*phox*^ to irradiation generating oxygen radicals, with a presumed effect on cysteines, inhibits oxidase assembly but was without effect on the assembled complex (Ostuni et al., 2010).

An important prerequisite for binding of p67^*phox*^ to Nox2 was the presence of a disulfide bond between cysteines 369 and 371. The characteristics of this bond are critical for binding, as shown by the requirement for cysteines to be separated by one non-cysteine residue and for their presence in the same molecule. Contrary to expectations, replacing the native CGC triad in the Nox2 peptides with the PDI (CGHC) or thioredoxin (CGPC) catalytic motifs did not result in enhanced binding. The requirement for a disulfide bond in Nox2 peptides for binding of p67^*phox*^ was one of the first indicators for a PDI-like function of Nox2, in accordance with the thiol—disulfide exchange reactions catalyzed by canonical PDIs (Ellgaard and Ruddock, 2005; Appenzeller-Herzog and Ellgaard, 2008). This raises the issue of the physiological mechanism to take place in the intact phagocyte responsible for the generation of the disulfide bond, when taking into account the fact that the cytosolic environment of the DHR of Nox2 is a reducing one. Also, such bond has to return to the dithiol state in order to assure the reversibility of the Nox2—p67^*phox*^ link. The stability of this link is a yet unsolved issue and depends on the conditions leading to the activation of the oxidase (van Bruggen et al., 2004; Tlili et al., 2012).

We can envisage several mechanisms for the oxidation of cysteines in Nox2. The oxidizing agent might be H_2_O_2_ derived by dismutation of O^·−^_2_ generated by the oxidase itself, leaking into the cytosolic milieu of the DHR of Nox2 (Enyedi et al., 2013). Another possibility is the intercalation of the CGC triad into the redox cascade of Nox2, from NADPH, via FAD/FADH_2_, to heme (Cross and Segal, 2004). An additional hypothetical mechanism is oxidation by an exogenous PDI in the oxidized form or by another thiol oxidase. Paes et al. (2011) indeed showed that addition of oxidized PDI enhanced oxidase activation in a cell-free system.

The involvement of a PDI in the assembly of the NADPH oxidase was championed by the group of F. Laurindo. They provided extensive experimental evidence for a role of a PDI in the regulation of NADPH oxidase activity in vascular smooth muscle and endothelial cells, suggesting a role in the stabilization of oxidase subunits assembly (Laurindo et al., 2008). A more specific role in the activation of phagocyte oxidase activation was also described, expressed in the ability of exogenous oxidized PDI to enhance and of reduced PDI to diminish oxidase activation *in vitro* (Paes et al., 2011). A peptide comprising the CGHC PDI catalytic motif inhibited oxidase activation and PDI–p47^*phox*^ and PDI–p22^*phox*^ associations were demonstrated (Santos et al., 2009; Paes et al., 2011).

We propose that, independently of the regulation of Nox2-dependent oxidase activity by an exogenous PDI, the DHR of Nox2 itself functions as an intrinsic PDI. The evidence for this is as follows:

1. By assessing the binding of p67^*phox*^ to seven NusA-Nox2 fusion proteins, serially truncated at the N-terminus of the Nox2 moiety, we found that binding required the presence of the CGC triad.
2. Fusion protein NusA-Nox2(357–570), which comprises the CGC triad, expressed dose-dependent PDI reductase activity.
3. Truncation of the Nox2 moiety in the NusA-Nox2 fusion protein C-terminal to the CGC triad or mutating C369 and C371 to R, resulted in the loss of PDI activity.
4. The PDI activity of NusA-Nox2 was suppressed by PAO.
5. A comparison of the sequence of the DHR of human Nox2 with several PDIs, revealed three small regions of homology with PDIA3.
6. Two monoclonal anti-Nox2 antibodies, with epitopes corresponding to two regions of Nox2/PDIA3 similarity, reacted with PDIA3 but not with PDIA1.
7. One polyclonal anti-PDIA3 antibody (but not an anti-PDIA1 antibody) reacted strongly with recombinant NusA-Nox2.
8. p67^*phox*^(1–212) in which all four cysteines were mutated to serines lost the ability to bind to a Nox2 peptide comprising the CGC triad and its capacity to activate the oxidase was impaired.

PDIA3 (also known as ERp57 or GRp58) is a 505-residues long protein, with a molecular mass of 56.78 kDa, found in the endoplasmic reticulum but also on the cell surface, plasma membrane, nucleus, cytosol, and in the secreted form (Frickel et al., 2004; Coe and Michalak, 2010). Its structure is similar to other PDIs (33% overall identity), with two catalytic domains (*a* and *a'*) comprising the CGHC motif and disulfide reduction potentials of −167 and −156 mV, respectively. It catalyzes disulfide reduction, dithiol oxidation, and disulfide isomerization. Its reductase activity was reported to be 20 times less efficient than that of PDIA1, using the insulin assay (Frickel et al., 2004); this is in disagreement with our finding, based on the DE-GSSG reduction assay, of a 30-fold higher activity of PDIA3 compared to PDIA1. PDIA3 promotes folding of glycoproteins in the endoplasmic reticulum and is a key component of the MHC-I peptide loading complex (Purcell and Elliot, 2008). A newly discovered role is that of a GDP dissociation inhibitor for the Ras family small GTPase Ra1A (Brymora et al., 2012).

The PDI reductase activity of NusA-Nox2(357–570) is much lower than that of recombinant PDIA1 and PDIA3, when compared by the DE-GSSG reduction assay. The reason for this might be the difference between the catalytic motifs (CGC, in Nox2, and CGHC, in most PDIs), the presence of two catalytic motifs in most PDIs, or the lesser affinity of Nox2 for the DE-GSSG substrate. Our results do not allow the allocation of functional roles to regions of homology between Nox2 and PDIA3. An evolutionary link between the two proteins is also unlikely. The most conservative interpretation is to look upon Nox2 as a relative of the large family of thiol-disulfide oxidoreductases, characterized by a CXXC motif (reviewed in Sevier and Kaiser, 2002).

In conclusion, we have described a rather unique situation, in which one protein (Nox2) mimics the enzymatic function of a seemingly unrelated protein (PDIA3), with which it shares a similar catalytic motif and minor sequence homology. We plan to expand this study by identifying the individual cysteines in p67^*phox*^ involved in binding to Nox2 and by designing NADPH oxidase inhibitors focused on the Nox2 CGC - p67^*phox*^ interaction.

## Authors contributions

Edna Bechor, Iris Dahan, Tanya Fradin, Yevgeny Berdichevsky, Anat Zahavi, Aya Federman Gross, Meirav Rafalowski and Edgar Pick were involved in the design and performance of the experiments and in the analysis of the results. Edgar Pick planed the conceptual framework and wrote the manuscript, which was approved by all the authors.

### Conflict of interest statement

The authors declare that the research was conducted in the absence of any commercial or financial relationships that could be construed as a potential conflict of interest.

## Abbreviations

CGC: ^357^CysGlyCys^371^
CGD: chronic granulomatous disease
DE-GSSG: dieosin glutathione disulfide
DTT: dithiothreitol
DHR: dehydrogenase region
EC_50_: half maximal effective concentration
FPLC: fast protein liquid chromatography
GSG: glutathione disulfide
HMBA: 4-(hydroxymercuri)benzoic acid
IC_50_: half maximal inhibitory concentration
LiDS: lithium dodecyl sulfate
mBBr: monobromobimane
NEM: *N*-ethylmaleimide
O^·−^_2_: superoxide
PAO: phenylarsine oxide
PBS: phosphate buffered saline pH 7.3
PCMB: *p*-chloromercuribenzoate
PDI: protein disulfide isomerase
ROS: reactive oxygen species
*V*_max_: maximal velocity.
