# Supplementary material for: The dehydrogenase region of the NADPH oxidase component Nox2 acts as a protein disulfide isomerase (PDI) resembling PDIA3 with a role in the binding of the activator protein p67phox
Source: Front Chem. 2015 Feb 4;3:3. doi: 10.3389/fchem.2015.00003 (PMC4316792; doi:10.3389/fchem.2015.00003)
Supplement: Supplementary file 1 [file DataSheet1.DOC]

**SUPPLEMENTARY MATERIAL**

**CONSTRUCTION OF NusA-Nox2 FUSION PROTEINS**

Several fusion proteins were generated, joining residues 1-495 of NusA (Davis et al., 1999) with residues 290-570 of NOX2, or parts of it, in plasmid pET-43a (Novagen, EMD Chemicals, Merck KGaA, Darmstadt, Germany). NusA-Nox2 (290-570) gene fusion construct was generated by direct subcloning of cDNA coding for Nox2 cytosolic residues 290-570 from plasmid pGEX-4T3-NOX2(290-570) (a gift of the late Dr. G. Bokoch, Scripps Institute, La Jolla, CA, USA) into *Bam*HI-*Xho*I sites of plasmid pET-43a. This procedure resulted in construction of a fusion protein where the NusA moiety is placed at the N-terminus and is separated from the C-terminally placed cytosolic part of Nox2 by a 58 residues peptide containing a 6His tag. Gene fusions of NusA with cytosolic parts of NOX2 spanning residues 328-570, 357-570, 372-570, 387-570, 408-570, 444-570 and 462-570, where constructed by PCR with N-terminal Nox2 specific shortening primers and with C-terminal COLIDOWN primer (Novagen), applying plasmid pET-43a-NOX2(290-570) as a template. All N-terminal primers introduced the *Bam*HI site. The PCR fragments were digested with *Bam*HI and *Xho*I restriction enzymes and subcloned into the same sites of plasmid pET-43a. In order to generate a shorter version of NusA protein for control binding experiments, the NusA gene along with the gHis tag sequence was subcloned from plasmid pET-43a into *Nde*I-*Bam*HI sites of plasmid pET-11c (Novagen). The cloning procedure resulted in construction of NusA protein where the peptide tail is similar (with addition of 2 amino acids only) to that which separates the NusA and Nox2 moieties in the gene fusions described above. The Platinum® Pfx DNA polymerase (Invitrogen, Life Technologies, Carlsbad, CA, USA) was used in all PCRs. The reaction conditions were as follows: 94 °C for 2 min, followed by 30 cycles each of 94 °C for 15 s, 55 °C for 30 s, and 68 °C for 90 s, and finally, 68 °C for 5 min. Digestion by restriction endonucleases (New England Biolabs, Ipswich, MA, USA), DNA purification from agarose gel, and plasmid and DNA fragment purifications after enzymatic reactions, using HiYield Gel/PCR DNA fragments Extraction Kit, and HiYield Plasmid Mini Kit, respectively, both from RBC Real Biotech Corporation (Banqiao City, Taipei, Taiwan), and molecular cloning, were performed as recommended by the suppliers. Throughout the entire work, the correctness and integrity of NusA-Nox2 fusion genes were confirmed by DNA sequencing.
